# Characterization of APOBEC3 variation in a population of HIV-1 infected individuals in northern South Africa

**DOI:** 10.1186/s12881-018-0740-4

**Published:** 2019-01-19

**Authors:** Nontokozo D. Matume, Denis M. Tebit, Laurie R. Gray, Stephen D. Turner, David Rekosh, Pascal O. Bessong, Marie-Louise Hammarskjöld

**Affiliations:** 1 Myles H. Thaler Center for AIDS and Human Retrovirus Research, Department of Microbiology, Immunology and Cancer Biology, University of Virginia, Charlottesville, VA USA; 2 Department of Public Health Sciences and Bioinformatics Core, University of Virginia School of Medicine, Charlottesville, VA 22908 USA; 3 HIV/AIDS & Global Health Research Programme and Department of Microbiology, University of Venda, Thohoyandou, South Africa; 4 Global Biomed Scientific LLC, PO Box 2368, Forest, VA 24551 USA

**Keywords:** APOBEC3, Single nucleotide polymorphism, South Africa

## Abstract

**Background:**

The apolipoprotein B mRNA-editing enzyme, catalytic polypeptide-like 3 (APOBEC3) genes A3D, A3F, A3G and A3H have all been implicated in the restriction of human immunodeficiency virus type 1 (HIV-1) replication. Polymorphisms in these genes are likely to impact viral replication and fitness, contributing to viral diversity. Currently, only a few studies indicate that polymorphisms in the A3 genes may be correlated with infection risk and disease progression.

**Methods:**

To characterize polymorphisms in the coding regions of these APOBEC3 genes in an HIV-1 infected population from the Limpopo Province of South Africa, APOBEC3 gene fragments were amplified from genomic DNA of 192 HIV-1 infected subjects and sequenced on an Illumina MiSeq platform. SNPs were confirmed and compared to SNPs in other populations reported in the 1000 Genome Phase III and HapMap databases, as well as in the ExAC exome database. Hardy-Weinberg Equilibrium was calculated and haplotypes were inferred using the LDlink 3.0 web tool. Linkage Disequilibrium (LD) for these SNPS were calculated in the total 1000 genome and AFR populations using the same tool.

**Results:**

Known variants compared to the GRCh37 consensus genome sequence were detected at relatively high frequencies (> 5%) in all of the APOBEC3 genes. A3H showed the most variation, with several of the variants present in both alleles in almost all of the patients. Several minor allele variants (< 5%) were also detected in A3D, A3F and A3G. In addition, novel R6K, L221R and T238I variants in A3D and I117I in A3F were observed. Four, five, four, and three haplotypes were identified for A3D, A3F, A3G, and A3H respectively.

**Conclusions:**

The study showed significant polymorphisms in the APOBEC3D, 3F, 3G and 3H genes in our South African HIV1-infected cohort. In the case of all of these genes, the polymorphisms were generally present at higher frequencies than reported in other 1000 genome populations and in the ExAC exome consortium database .

**Electronic supplementary material:**

The online version of this article (10.1186/s12881-018-0740-4) contains supplementary material, which is available to authorized users.

## Background

The genes for the apolipoprotein B mRNA-editing enzyme catalytic polypeptide-like protein gene family (APOBEC3), a family of seven members (APOBEC3 A, B, C, D, F, G and H), are situated on human chromosome 22. The proteins encoded by these genes are cytidine deaminases that have been classified as restriction factors because of their role as innate immunity factors. They provide host cell defense against a diverse set of retroviruses, endogenous retroelements and DNA viruses, including human immunodeficiency virus (HIV) [1–3]. APOBEC proteins restrict HIV through deamination of cytosines in viral cDNA during reverse transcription, causing G-to-A hypermutations in the viral DNA product, which results in degradation and viral inhibition [4]. The Vif protein of HIV has evolved to counteract this restriction by binding to APOBEC proteins leading to proteasomal degradation.

One of the most studied APOBEC proteins and the first that was discovered to restrict HIV-1 replication is APOBEC3G. In the absence of the HIV-1 Vif protein, APOBEC3G is efficiently packaged into viral particles, causing restriction during reverse transcription. The gene was originally identified as an HIV restriction factor because its expression converted a T-cell line that could support the replication of an HIV lacking *vif* into one that had a non-permissive phenotype [1].

Three other members of the APOBEC family, APOBEC3D (A3D), APOBEC3F (A3F), and APOBEC3H (A3H) can also be packaged into HIV particles and inhibit viral replication, when stably expressed in human T-cell lines [5]. Endogenous A3D and A3F combine to generate the 5′-GA-to-AA mutation pattern observed in *vif*-negative HIV grown in the non-permissive T-cell line CEM2n [6, 7]. Of the seven different human haplotypes of APOBEC3H, only hapII, hapV and hapVII are stable at the protein level and capable of HIV restriction [8–12].

Several APOBEC3 (A3D, A3F, A3G and A3H) genes are known to possess common polymorphisms that render them defective with reduced antiviral activity and increased sensitivity to HIV-1 Vif [5, 13–16]. The genetic associations between natural polymorphisms in APOBEC genes and the ability of the resulting proteins to restrict HIV and the contribution of polymorphisms to overall HIV diversity and disease progression have not received widespread attention. Polymorphisms in APOBEC genes could also play a significant role in HIV-1 evolution and diversity, especially in African populations, where the prevalence of HIV-1 is still increasing.

African populations are characterized by a high level of genetic diversity owing to a large number of variable genes and alleles [17–20]. Patterns of genetic variation in the African population are influenced by a demographic history that includes changes in population size, admixture and locus-specific forces such as natural selection, recombination and mutation. Genetic studies of structural variation of genes across ethnically diverse populations have been conducted [21]. Many population genetic studies of African populations are based on analysis of genetic markers genotyped in a small number of people in selected populations, in projects such as the 1000 Genomes Project (2010) and the International Haplotype Map (HapMap) Project [22–24]. Although these projects are valuable in their description of the overall human genetic diversity, they are limited in their coverage of African populations [25]. Thus, it is important to continue to add information about African populations that are underrepresented in human genomic studies, such as the South African population.

South Africa embodies a rich collection of ethnic backgrounds in addition to the more recent Caucasian immigrants. The major ethnic groups include the Bapedi, Basotho, Ndebele, Swati, Tsonga, Tswana, Xhosa, Venda and Zulu. The genetic substructure of these populations has been assessed by studying the Y-chromosome and autosomal DNA resulting into a cluster of three specific groups: Tswana/Sotho, Nguni and Venda [26, 27]. It is of clear interest to characterize the APOBEC3 gene polymorphisms existing in these various populations, since they may play a crucial role in the restriction and evolution of HIV-1.

In the current study, we characterized the genetic variability within the coding regions of A3D, A3F, A3G and A3H to document the level of diversity in samples obtained from HIV-1 positive individuals attending three HIV clinics in the Limpopo Province of Northern South Africa.

## Methods

### Study population and DNA extraction

The study population was comprised of a total of 192 HIV-1 positive individuals from several ethnic groups (Venda, Bapedi, Tswana, Tsonga and Swati) who presented for routine care in clinics and hospitals in the Waterberg and Vhembe districts of the Limpopo province in Northern South Africa. There were 116 females and 76 males with an age range from 4 to 98 years and their viral load and CD4+ cell count ranged from < 20 to 623,250 copies/ml and 5 to 1353 cells/μl, respectively (Additional file 1: Table S1). These individuals were recruited from July 2013 to December 2015. DNA was extracted from peripheral blood mononuclear cells (PBMC), using the QIAamp DNA blood mini kit (Qiagen) according to the manufacturer’s instructions.

### Primer design

Primers to amplify the four APOBEC3 genes (A3D, A3F, A3G, and A3H) were designed using Geneious® 8.1.5 software (Biomatters, Inc.). A nested PCR strategy was used to amplify each APOBEC gene. The outer primer set was designed to flank and amplify a long gene fragment in the 1st polymerase chain reaction (PCR), while two sets of primers were designed to amplify two fragments of each gene in a nested PCR using the 1st round PCR product as the template (Table 1). The primer sets were chosen using the information for the A3D, A3F, A3G and A3H genes in the Ensembl Genome Browser (ENSG00000243811, ENSG00000128394, ENSG00000239713 and ENSG00000100298).

**Table 1:** List of APOBEC3 primers designed; primer name, sequence and product size are indicated

Name	Sequence (5′-3′)	Product size
A3D (12.1 kb)		
A3D Forward primer	AGGAAGCCTCGCTCTCTCA	12,069 bp
A3D Forward primer	CAGGCAGGGTCTTGATCTGT	
A3D Amplicon 1F	AAAAAGAGGGAGACTGGGACAAGCGTATCTAAGA	4,300 bp
A3D Amplicon 1R	GAGTGTGGGTGAGGGGGTGTAACCATGAG	
A3D Amplicon 2F	AGCTAGGAGAGGTCACCCTG	3,188 bp
A3D Amplicon 2F	CAGGAGGCTAGAAGAGACAGACCATGAGGC	
A3F (13.31 kb)		
A3F 1st round f	ACCAGAAAGAGGGTGAGAGACTGAGGAAGATAAAG	13,142 bp
A3F 1st round rv	AGCCATTTATTGCAGAAGCTATGGATAAAGCTGGT	
A3F Amplicon 1 f	ACCAGAAAGAGGGTGAGAGACTGAGGAAGATAAAG	4,918 bp
A3F Amplicon 1 rv	GGGTGAGGGGTGTAACCATG	
A3F Amplicon 2 f	TTCAGAAACCCGATGGAGGC	4,478 bp
A3F Amplicon 2 rv	AGCCATTTATTGCAGAAGCTATGGATAAAGCTGGT	
A3G (10.74 kb)		
A3G 1st round f	TGTTAACCAGAGGCTGCTCTTCCCAGG	11,852 bp
A3G 1st round rv	TCCCTGGGACTCAGCTCC	
A3G Amplicon 1 f	ATTTGTCCCCAGCTCTGTGG	3,231 bp
A3G Amplicon 1 rv	AGAGGACCTGGTCTGGAACA	
A3G Amplicon 2 f	CAAGGGAGGAAGCGTGGAG	3,908 bp
A3G Amplicon 2 rv	TGCATTGCTTTGCTGGTGTC	
A3H (6.8 kb)		
APOBEC3 H forward primer full length	TCTGTTGCACAGAAACACGATGG	3522bp
APOBEC3 H reverse primer full length	CAACTGACATGCCCCAGGG	
APOBEC3 H forward primer Exon2 (A3HfE2)	TCTGTTGCACAGAAACACGATGG	452bp
APOBEC3 H Reverse primer Exon 2(A3HrE2)	TTCCCGAAGTAGTGACTGAGC	
APOBEC3 H forward primer Exon 3 &4(A3HfE3/4	GCCACGCACTAGAAAGTTCAC	934bp
APOBEC3 H Reverse primer Exon 3&4(A3HrE3/4)	ACAGTGCCTCACCTTTATCC	

### Polymerase chain reaction (PCR) to amplify A3D, A3F, A3G and A3H genes

The Takara (LA) PCR Kit Ver. 2.1 for long DNA fragments amplification (Clontech) was used to amplify the complete 12.16 kb A3D, 13.31 kb A3F, 10.74 kb of A3G, and 6.8 kb A3H genes in a 1st round PCR reaction using genomic patient DNA. The 1st round primary PCR products were then used as templates in “nested” PCR reactions to generate shorter PCR products/ All the PCR reactions contained: 1X PCR Mg^2+^ plus buffer, 400 μM dNTPs, 0.2 μM of each primer (Table 1) and 1.25 units of LA Taq high fidelity polymerase in a total volume of 20 μl. The following cycling conditions were used for all PCR reactions: Initial denaturation at 94 °C for 1 min, 30 cycles of denaturation at 98 °C for 10s, annealing at temperatures varying from 53 °C to 68 °C for 15 min (depending on primers) and extension at 72 °C for 10 min. Final amplicons were purified using AMpure XP beads (Beckman Coulter) and quantified using a Qubit 3.0 Fluorometer with the dsDNA HS kit (Invitrogen). Equimolar concentrations of the two shorter amplicons generated for each gene were pooled and normalized to 1 ng using 10 mM Tris elution buffer.

### Fragmentation, tagmentation and addition of Illumina indices

Purified Tn5 transposase enzyme was used to fragment about 1-10 ng of DNA amplicons to sizes ranging from 35 bp to 700 bp, tagged with sequencing adaptors, in a manner similar to the protocol used in the Illumina Nextera Kit. The reaction mixture contained: 4 μl tagmentation buffer (5X TAPS-DMF), 1-5 μl Tn5 transposase (1X-5X) and 1-10 ng DNA, with an addition of nuclease free water to add up to a final volume of 20 μl. The reaction was performed at 55 °C for 5 min. The Tn5 transposase enzyme was produced and characterized in the University of Virginia laboratory, using published protocols [28]. Following this step, unique Illumina dual-index barcodes (index1 (i7) and index 2 (i5)) were added to each sample in a short PCR of 12 cycles, followed by a second AMpure XP bead purification, generating 300-500 bp indexed fragments for sequencing. Using the full complement of Nextera XT indices, up to 96 individual samples were pooled for each run.

### Library normalization, pooling and sequencing

After purification, libraries were size-verified using a bioanalyzer 2100 with a High Sensitive DNA assay kit (Agilent Genomics), quantified and normalized to a concentration of 4 nM each. The normalized libraries were then pooled, and denatured into single strands. For good cluster generation, 1.8pM of the pooled library spiked with 25–30% PhiX was then loaded into the sequencing cartridge. Biological sample sheets were created in Basespace by labeling each sample with the appropriate index and setting up a sequencing run for the MiSeq. Each run generated approximately 25 million reads/sequences per sample.

### Demultiplexing and sequence quality control evaluation

Sequences were demultiplexed automatically on the MiSeq as part of the data processing steps and ends pairing. FASTQ files were generated for each sample representing the two paired-end reads. Sequence quality was validated using the Galaxy NGS platform Quality Control tools for sequence manipulation which includes the fastQC program.

### Sequence filtering, trimming, mapping and variant calling

Sequencing data quality, including the duplication rate, percent GC, and read quality was assessed by quality control tools for high throughput sequencing data [29, 30]. After filtering low coverage samples, reads were aligned against the human genome with BWA-MEM [31]. Alignments were sorted, marked for duplicates, and indexed using SAMtools [32]. Variants were called using Freebayes, a Haplotype-based tool to detect variants using short-read sequencing data [33]. Variant calls were normalized and decomposed with vt, a unified representation of genetic variants, and functionally annotated using SnpEff, a program for annotating and predicting the effects of single nucleotide polymorphisms [34]. Comprehensive annotation and prioritization was performed using the GEMINI framework for Integrative Exploration of Genetic Variation and Genome Annotations [35]. All further data manipulation and analysis was performed using R, a Language and Environment for Statistical Computing [36].

### Statistical analysis

#### Hardy-Weinberg equilibrium (HWE) and allele frequency comparisons

All variant loci detected within the coding regions of these genes were tested for deviation from the Hardy-Weinberg Equilibrium (HWE) using an excel HWE calculator and chi-squared test with *P* < 0.05 showing non-consistency with HWE [37]. To statistically assess the differences between allele frequencies in our SA population and other populations, a Fisher’s exact test was conducted using an online Graphpad QuickCalcs tool (https://www.graphpad.com/quickcalcs/contingency1.cfm), with the exception of the comparison with the large ExAC exome population, where a chi-squared test was used.

#### Pairwise linkage disequilibrium (LD) and haplotype assignment

Pairwise linkage disequilibrium (LD) analysis between the SNPs in each gene was performed to test if they were in LD in linkage disequilibrium in the African population from the 1000 Genome (1000G) project phase 3 (version 5), as well as in the entire 1000G population. This was done using the LDLink 3.0 web tool LDmatrix and LDpair modules (https://analysistools.nci.nih.gov/LDlink/?tab=home). This tool investigates patterns of linkage disequilibrium returning calculated D prime (D’), R squared (R^2^) and goodness-of-fit (chi-squared and *p*-values) to the variant rs number assigned by dbSNP that were used as input. Haplotypes for each APOBEC 3 gene were defined using the LDhap module, which calculates population specific haplotypes frequencies of all haplotypes observed for a list of query variants, using data from the 1000 Genome project phase 3 (version 5) [38]. The haplotypes present in each individual were then tallied from our sequence data, and the frequency of each haplotype within the population was calculated.

## Results

### Single nucleotide polymorphisms (SNPs), detection of indels and verification

There is limited availability of APOBEC3 gene sequences from African populations, and when sequencing has been performed, it has often been limited to A3G [39]. In this study, we applied next generation sequencing to determine variation in the coding exons of the APOBEC genes A3D, A3F, A3G and A3H in DNA from 192 HIV-1 positive individuals residing in the Limpopo province of northern South Africa. The proteins expressed from these genes have all been shown to be capable of HIV restriction [5]. APOBEC 3 variation in this region has not been reported previously.

#### APOBEC3D

The A3D gene is 12.1 kb long (Table 1) and has seven exons with exon 5 shown to display the most variation. Good quality A3D sequences after targeted DNA amplification of the exons were successfully obtained for 168/192 subjects. In the DNA from these 168 individuals, 8 nonsynonymous and 2 synonymous changes were identified when compared to the GRCh37 build of the human genome (Table 2). Of the 168 subjects analyzed, 48.8% (82/168) were identified with nonsynonymous or synonymous changes in many positions in the coding region of the A3D gene, while no changes were detected in the remaining 51.2% (86/168). These changes included several previously identified changes. There were no insertions or deletions observed in A3D in the sequenced samples. Variant R248K was the most frequent, observed in 20.8% (35/168) of the patients, with 2 homozygotes, followed by R97C that was found in 11,9% (20/168) with 1 homozygote Three variants, R6K, L221R, and T238I, that have not been reported elsewhere, were observed as heterozygotes in 10.1, 1.8 and 4.8% of the patients respectively. No variants deviated from HWE (Table 2). Linkage disequilibrium (LD) values for the four SNPs with known allele frequencies in the 1000 genome populations were calculated using the total 1000G population, as well as the AFR group (see Additional file 2: Table S2). Most of the variants are not in LD (cut off > 0.1) in these populations, except for R248K and T316 T that are in marginal LD (D’ = 1, R^2^ = 0.122) in the overall, but not in the AFR group.

**Table 2:** APOBEC 3D, 3F, 3G and 3H nonsynonymous and synonymous changes, genotypes, amino acid position and change in the protein, frequencies and Hardy Weinberg Equilibrium calculations from the study population

Amino acid change and variant ID	Type of change	Genotypes nt # in CDS	Exon	Frequencies (%)	Hardy Weinberg equilibrium
APOBEC 3D nonsynonymous changes (*n* = 168)					
**R6K (**NI)	A**G**A➔A**A**A Transition	17G/G 17G/A	1	151 (89.9) 17 (10.1)	*P*-value = 0.49 X^2^ = 0.48
**R97C** (rs75858538)	**C**GC➔**T**GC Transition	289 C/C 289 C/T 289 T/T	1	148 (88.1) 19 (11.3) 1 (0.6)	*P*-value = 0.65 X^2^ = 0.20
**L221R** (NI)	C**T**G➔C**G**G Transition	662 T/T 662 T/G	5	165 (98.2) 3 (1.8)	*P*-value = 0.91 X^2^ = 0.01
**C224Y** (rs772893975)	T**G**T➔T**A**T Transition	671G/G 671G/A	5	161 (95.8) 7 (4.2)	*P*-value = 0.78 X^2^ = 0.076
**T238A** (rs201709403)	**A**CA➔**G**CA Transition	712A/A 712A/G	5	154 (91.7) 14 (8.3)	*P*-value = 0.57 X^2^ = 0.32
**T238I** (NI)	A**C**A➔A**T**A Transition	713C/C 713C/T	5	160(95.2) 8(4.8)	*P*-value = 0.75 X^2^ = 0.10
**R248K** (rs61748819)	A**G**G➔A**A**G Transition	743 G/G 743 G/A 743 A/A	5	133 (79.2) 33 (19.6) 2 (1.2)	*P*-value = 0.98 X^2^ = 0.001
**C320Y** (rs61999342)	T**G**C➔T**A**C Transition	959 G/G 959 G/A	6	167 (99.4) 1 (0.6)	*P*-value = 0.97 X^2^ = 0.001
APOBEC3D synonymous changes					
**L221 L** (rs769426665)	CT**G** ➔CT**C** Transversion	663G/G 663G/C	5	165 (98.2) 3 (1.8)	*P*-value = 0.91 X^2^ = 0.01
**T316 T** (rs184448269)	AC**C**➔AC**T** Transition	948 C/C 948 C/T	6	161 (95.8) 7 (4.2)	*P*-value =0.78 X^2^ = 0.08
APOBEC 3F nonsynonymous changes (*n* = 154)					
**R48P** (rs35053197) (in isoform 201 and 202)	C**G**T➔C**C**C Transversion	143 G/G 143 G/C	2	142 (92.2) 12 (7.8)	*P*-value = 0.61 X^2^ = 0.25
**A78V** (rs5750728) (only in isoform 201)	G**C**C➔G**T**C Transition	233 C/C 233 C/T 233 T/T	4	95 (61.7) 56 (36.4) 3 (1.9)	*P*-value = 0.10 X^2^ = 2.64
**I87L** (rs146543452) (only in isoform 201)	**A**TC➔**C**TC Transversion	259 A/A 259 A/C	4	153 (99.4) 1(0.6)	*P*-value = 0.97 X^2^ = 0.002
**Q87L** (rs114704208) (only in isoform 202)	C**A**G➔C**T**G Transversion	260 A/A 260 A/T	3	145 (94.2) 9 (6.8)	*P*-value = 0.71 X^2^ = 0.14
**A108S** (rs2020390) (only in isoform 201)	**G**CT➔**T**CT Transversion	322 G/G 322 G/T 322 T/T	4	54 (35.1) 84 (54.5) 16 (10.4)	*P*-value = 0.04 X^2^ = 4.02
**V231I** (rs2076101) (only in isoform 201)	**G**TC➔**A**TC Transition	691 G/G 691 G/A 691 A/A	5	122 (79.2) 30 (19.5) 2 (1.3)	*P*-value = 0.92 X^2^ = 0.01
**Y307C** (rs12157816) (only in isoform 201)	T**A**C➔T**G**C Transition	920 A/A 920 A/G	6	139 (90.3) 15 (9.7)	*P*-value = 0.52 X^2^ = 0.40
APOBEC3F synonymous changes					
**I117I** (NI) (only in isoform 201)	AT**C**➔AT**T** Transition	351 C/C 351 C/T	4	152 (98.7) 2 (1.3)	*P*-value =0.94 X^2^ = 0.007
**S118S** (rs35928287) (only in isoform 201)	TC**C**➔TC**T** Transition	354 C/C 354 C/T	4	113 (73.4) 41 (26.6)	*P*-value =0.06 X^2^ = 3.63
**R143R** (rs4821862) (only in isoform 201)	CG**C**➔CG**T** Transition	429 C/C 429 C/T 429 T/T	4	19 (12.3) 91 (59.1) 44 (28.6)	*P*-value = 0.01 X^2^ = 7.04
**Y196Y** (rs765418322) (only in isoform 201)	TA**T**➔TA**C** Transition	588 T/T 588 T/C 588 C/C	4	126 (81.8) 24 (15.6) 4 (2.6)	*P*-value = 0.04 X^2^ = 4.09
**S229S** (rs549550231) (only in isoform 201)	TC**A**➔TC**G** Transition	687 A/A 687 A/G	5	152 (98.7) 2 (1.3)	*P*-value = 0.94 X^2^ = 0.007
**E245E** (rs113109079) (only in isoform 201)	GA**G**➔GA**A** Transition	735 G/G 735 G/A 735 A/A	5	146 (94.8) 7 (4.5) 1 (0.7)	*P*-value = 0.01 X^2^ = 6.09
**S327S** (rs35895636) (only in isoform 201)	TC**C**➔TC**T** Transition	981 C/C 981 C/T 981 T/T	5	128 (83.2) 23 (14.9) 3 (1.9)	*P*-value = 0.12 X^2^ = 2.39
APOBEC 3G nonsynonymous changes (*n* = 165)					
**H186R** (rs8177832)	C**A**C➔C**G**C Transition	557 A/A5 557 A/G 557 G/G	4	63 (38.2) 82 (49.7) 20 (12.1)	*P*-value = 0.39 X^2^ = 0.73
**R256H** (rs17000736)	C**G**C➔C**A**C Transition	767 G/G 767 G/A	6	161(97.6) 4 (2.4)	*P*-value = 0.87 X^2^ = 0.02
**Q275E** (rs17496046)	**C**AG➔**G**AG Transversion	823 C/C 823 C/G 823 G/G	6	111 (67.3) 49 (29.7) 5 (3.0)	*P*-value = 0.88 X^2^ = 0.02
**G363R** (rs148267053)	**G**GA➔**A**GA Transition	1087 G/G 1087 G/A	7	148 (89.7) 17 (10.3)	*P*-value = 0.49 X^2^ = 0.49
APOBEC3G synonymous changes					
**S60S** (rs112603901)	TC**C**➔TC**T** Transition	180 C/C 180 C/T	3	147 (89.1) 18 (10.9)	*P*-value =0.46 X^2^ = 0.55
**A109A** (rs375760983)	GC**C**➔GC**T** Transition	327 C/C 327 C/T	3	164 (99.4) 1 (0.6)	*P*-value =0.97 X^2^ = 0.002
**F119F** (rs5757465)	TT**T**➔TT**C** Transition	357 T/T 357 T/C	3	164 (99.4) 1 (0.6)	*P*-value =0.97 X^2^ = 0.002
**L371 L** (rs11545130)	**C**TG➔**T**TG Transition	1111 C/C 1111 C/T	7	158 (95.8) 7 (4.2)	*P*-value =0.78 X^2^ = 0.08
APOBEC 3H nonsynonymous changes (*n* = 133)					
**N15Δ** (rs140936762)	**-CAA** Deletion	45 CAA/CAA 45 CAA/ Δ 45 Δ / Δ	1	29 (21.8) 49 (36.8) 55 (41.4)	*P*-value = 0.001 X^2^ = 10.25
**R18L** (rs139293)	C**G**C➔C**T**C Transversion	53 G/G 53 G/T 53 T/T	1	112 (84.2) 15 (11.3) 6 (4.5)	*P*-value = 0.00 X^2^ = 15.9
**G105R** (rs139297)	**G**GC➔**C**GC Transversion	313 G/G 313 G/C 313 C/C	2	1 (0.8) 4 (3.0) 128 (96.2)	*P*-value = 0.00 X^2^ = 13.4
**K121E** (rs139298)	**A**AG➔**G**AG Transition	361 A/A 361 A/G 361 G/G	2	1 (0.8) 6 (4.5) 126 (94.7)	*P*-value = 0.01 X^2^ = 6.9
**K140E** (rs139300)	**A**AG➔**G**AG Transition	418 A/A 418 G/G	2	0133 (100)	*P*-value = N/A X^2^ = N/A
**E178D** (rs139302)	GA**G**➔GA**C** Transversion	534 G/G 534 G/C 534 C/C	3	3 (2.2) 11 (8.3) 119 (89.5)	*P*-value = 0.00 X^2^ = 12.7
APOBEC3H synonymous changes					
**T43 T** (rs139294)	AC**G**➔AC**C** Transversion	129 G/G 129 G/C 129 C/C	1	6 (4.5) 7 (5.3) 120 (90.2)	*P*-value =0.00 X^2^ = 48.4

The following Ensembl transcripts were used for aa positions in the CDS:

A3D: APOBEC3D-201 ENST00000216099.12 (386aa)

A3F: APOBEC3F-201 ENST00000308521.9 (373aa) and APOBEC3F-202 ENST00000381565.2 (101aa)

A3G: APOBEC3G-201 ENST00000407997.3 (384aa)

A3H: APOBEC3H-204 ENST00000442487.7 (183aa)

NI = Not Identified Previously;

Nucleotide change in the codon is shown in bold

CDS = coding sequence

#### APOBEC3F

The A3F gene is 13.3 kb long (Table 1). Two major transcript isoforms have been described for this gene (APOBECF-201 and APOBECF-202 in ENSEMBL). These contain seven and three exons, respectively and share one exon (exon 2). The most variation has been observed in APOBEC-201 exon 4. The A3F exons were all successfully amplified and sequenced from a total of 154/192 subjects. Synonymous or nonsynonymous changes were observed in 98.1% (151/154) of the subjects, while 1.9% (3/154) had no change relative to the GRCh37 human genome build (Table 2). In the 154 samples successfully sequenced, there were seven nonsynonymous changes (R48P, A78V, I87L, Q87L, A108S, V231I and Y307C) and seven synonymous changes (I117I, S118S, R143R, Y196Y, S229S, S327S and E245E). A78V and A108S were the most frequent nonsynonymous changes in A3F, found in 38.3 and 64.9% and of the subjects respectively (Table 2). A few of these variants (A108S, R143R, Y196Y and E245E), deviated from the HWE (*P*-values < 0.05). The synonymous I117I mutation has not been reported previously. No insertions or deletions were observed for A3F in the sequenced samples. LD values for rs variants with known allele frequencies in the 1000G database for the overall and AFR group are shown in Additional file 3: Table S3. As can be seen in the table, several of the A3F variants, are in strong LD with each other in these populations.

#### APOBEC3G

The A3G gene was the first APOBEC3 gene described as encoding an HIV restriction factor and it remains the most studied. The gene is 10.7 kb and has 8 exons (Table 1). We successfully amplified A3G from 165/192 subjects. A total of four nonsynonymous (H186R, R256H, Q275E and G363R) and four synonymous changes (S60S, A109A, F119F and L371 L) were observed in A3G with the most frequent being H186R (61.8%) and Q275E (32.7%), (Table 2). All of these variants have been described previously. In total, nonsynonymous or synonymous changes were observed in 91.5% (151/165) of our patients, whereas 8.5% (14/165) had no changes relative to the reference GRCh37 human genome. There were no insertions or deletions observed in this gene. No variants deviated from HWE (Table 2). LD values could be calculated for all of these variants with the exception of A109A (Additional file 4: Table S4), which had a very low frequency in our population.. Most of the variants are not in LD, but H186R and Q275E are in marginal LD (D’ = 1, R^2^ = 0.108) in the AFR group.

#### APOBEC3H

A3H is the shortest, but most polymorphic of the APOBEC3 genes we analyzed. It is 6.8 kb in length (Table 1) and contains 5 exons, with the most variation in exons 1, 2 and 3. We observed nonsynonymous or synonymous changes in all the study subjects that we obtained sequences from (133/192). We found 6 nonsynonymous changes (N15Δ, R18L, G105R, K121E, K140E and E178D) and one synonymous change (T43 T) (Table 2). The N15Δ deletion was the only deletion observed and it occurred in 104 of 133 subjects (78.2%) either in a homozygous (49) or heterozygous (55) form. No insertions were found. The T43 T, G105R, K121E, K140E and E178D variants occurred mostly as homozygous forms in 95.5–100% of all subjects (Table 2). The K140E variant is also present as a homozygous variant in 100% in the 1000G and ExAC databases (see Table 4) and is thus likely to represent a sequencing error in the reference genome or an extremely rare variant in the human population. All of the other A3H variants deviated significantly from the HWE (*P*-value < 0.05), (Table 2). All of the variants with the exception of K140E (where this could not be calculated) are in LD in the overall 1000G population and many are in LD also in the AFR group (Additional file 5: Table S5).

### Determination of APOBEC 3 haplotypes

In order to better understand the A3 genetic changes observed in each subject, all clusters of variation within the genes were assigned into haplotypes as described in materials and methods and their frequencies calculated. These haplotypes were classified as either confirmed or unconfirmed based on the number of heterozygous variants. This classification was necessary due to the fact that the NGS reads were short and thus in many cases we could not determine if SNPs occurred on the same chromosome (Table 3). Nonsynonymous variants were considered and their genotypes (homozygous or heterozygous) were indicated. Low frequency variants (MAF < 5%) were excluded from the haplotype assignment. Comparisons were made to the GRCh37 human genome whose combinations are represented as haplotypes in A3D, A3F and A3G (Table 3). We identified four confirmed haplotypes for A3D, four confirmed haplotypes for A3F and four confirmed haplotypes for A3G (Table 3). It is worth noting that only haplotypes for A3G and A3H have been described previously [12, 15, 40, 41]. In the case of A3H, there are seven well characterized and six additional haplotypes that were recognized more recently. The seven well characterized haplotypes of A3H were recently described as having an impact on the genetic diversity of HIV-1 Vifs in the global pandemic [12, 15, 16]. All of the known A3H haplotypes (I-XIII) are combinations of 5 nucleotide changes located in exons 2, 3 and 4. Haplotypes II, V, and VII have been termed stable, because of the observed relatively long half-lives of the encoded proteins, enabling them to restrict HIV-1. Four of the haplotypes (I, III, IV, VI) have been termed unstable, since the encoded protein half-lives have been shown to be short, resulting in complete loss of the ability to restrict HIV [12, 39]. In our subjects, we identified 4 haplotypes for A3H: the stable haplotype II (15 N, 18R, 105R, 121E 178D), haplotype III (15Δ, 18R, 105R, 121E, 178D), haplotype IV (15Δ, 18 L, 105R, 121E, 178D) and haplotype X (15 Δ, 18R, 105R, 121E, 178E) (Table 3) [11, 12, 39]. Haplotypes III, IV and X all have the amino acid 15 deletion, known to make the Apobec 3H protein unstable. From the data in Table 2 and this haplotype analysis we can conclude that 41.4% of our patient population cannot express any stable ApoBec3H proteins and thus lack the ability to restrict HIV using Apobec 3H.

**Table 3:** Haplotypes frequencies for A3D, A3F, A3G and A3H

Variation (amino acid and its position)	Frequency (%)	Haplotypes within individuals
Confirmed APOBEC3D Haplotypes (*n* = 168)		
97R, 238 T, 248R	88 (52.3)	i/i
**97C**, 238 T, 248R	1 (0.6)	ii/ii
**97C(het)**, 238 T, 248R	18 (10.7)	i/ii
97R, **238A(het)**, 248R	10 (6)	i/iii
97R, 238 T, **248 K**	1 (0.6)	iv/iv
97R, 238 T, **248 K(het)**	27 (16)	i/iv
Minor variant frequency < 5%	7 (4.1)	Not assigned
Others^a^	16 (9.7)	Not assigned
Unconfirmed APOBEC3D Haplotypes		
**None**		
Confirmed APOBEC3F Haplotypes (*n* = 154)		
48R, 78A, 87I, 108A, 231 V, 307Y	5 (3.2)	i/i
48R, 78A, 87I, **108S**, 231 V, 307Y	2 (1.3)	iii/iii
48R, 78A, 87I, **108S(het)**, 231 V, 307Y	32 (20.8)	i/iii
48R, 78A, 87I, 108A, 231 V, **307C(het)**	5 (3.2)	i/iv
48R, **78 V**, 87I, **108S**, 231 V, 307Y	1 (0.6)	vi/vi
48R, **78 V (het)**, 87I, **108S**, 231 V, 307Y	2 (1.3)	vi/iii
Minor variant frequency < 5%	5 (3.2)	Not assigned
Others^a^	51 (33.1)	Not assigned
Unconfirmed APOBEC3F Haplotypes		
48R, **78 V (het)**, 87I, **108S (het)**, 231 V, 307Y	21 (13.6)	Not assigned
48R**, 78 V (het)**, 87I, **108S (het)**, **231I (het)**, 307Y	14 (9.1)	Not assigned
48R**, 78 V (het)**, 87I, **108S**, **231I (het)**, 307Y	8 (5.2)	Not assigned
48R**, 78 V (het)**, 87I, **108S (het)**, **231I**, 307Y	2 (1.3)	Not assigned
**48P (het)**, 78A, 87I, **108S (het)**, 231 V, 307Y	6 (3.9)	Not assigned
Confirmed APOBEC3G Haplotypes (*n* = 165)		
186H, 275Q, 363G	5 (3.1)	i/i
**186R**, 275Q, 363G	20 (12.1)	ii/ii
**186R (het)**, 275Q, 363G	43 (26.1)	i/ii
186H, **275E**, 363G	3 (1.8)	iii/iii
186H, **275E(het)**, 363G	22 (13.3)	i/iii
186H, 275Q, **363R(het)**	9 (5.5)	iv/iv
Minor variant frequency < 5%	11 (6.6)	Not assigned
Others^a^	52 (31.5)	Not assigned
Unconfirmed APOBEC3G Haplotypes		
**None**		
Confirmed APOBEC3H Haplotypes^b^ (*n* = 133)		
15 N, 18R, **105R**, **121E**, **178D**	38 (28.6)	ii/ii
**15Δ**,18R, **105R**, **121E**,**178E**	25 (18.8)	x/x
**15Δ(het)**, 18R, **105R**, **121E**, **178D**	36 (27.1)	ii/iii
**15Δ, 18 L**, **105R**, **121E**, **178D**	6 (4.5)	iv/iv
**15Δ, 18 L(het)**, **105R**, **121E**, **178D**	7 (5.3)	iv/iii
Other^a^	10 (7.4)	Not assigned
Unconfirmed APOBEC3H Haplotypes^b^		
**15Δ(het)**, 18R, **105R**, **121E(het)**, **178D**	4 (3)	Not assigned
**15Δ(het), 18 L(het)**, **105R**, **121E**, **178D**	7 (5.3)	Not assigned

**Bold** defines variants that are different from those listed in haplotype I in each gene

All variants marked by (het) are heterozygous. All others are homozygous

Haplotypes are called unconfirmed in our population due to more than 1 heterozygous SNP in the cluster

^a^Refers to the haplotypes with synonymous changes and those of novel SNPs (not reported on the dbSNP)

^b^A3H haplotypes were determined using previous classification from references [11, 12, 37]

### Allele frequencies and their comparison with other populations

We next compared the nonsynonymous and synonymous variant frequencies in the South African population in our study to previously reported variant frequencies in the following populations: African (AFR), East Asian (EAS), European (EUR), Ad Mixed American (AMR), and South Asian (SAS), as reported in the 1000 Genome Project phase III, the HapMap project (NCBI), the dsSNP database and the Ensembl genome browser. We also compared our allele frequencies to the ExAC consortium database that contains sequences from more than 60,000 individuals (Table 4).

**Table 4:** Comparison of A3D, A3F, A3G and A3H allele frequencies between our South African population (**SA**) and populations in the 1000 Genome Project including: East Asian (**EAS**), European (**EUR**), African (**AFR**), Ad Mixed American (**AMR**), South Asian (**SAS**), as well as data from the Exome Aggregation Consortium (**ExAC**)

Amino acid change and variant ID	Allele (2n)	SA (336)	EAS (1008)	EUR (1006)	AFR (1322)	AMR (694)	SAS (978)	ExAC (121412)
APOBEC 3D nonsynonymous allele frequencies (%)								
**R6K (**NI)	G (**R**) A (**K**)	94.9 5.1	NI	NI	NI	NI	NI	NI
**R97C** (rs75858538)	C (**R**) T (**C**)	93.8 6.2	100 0 (*P* < 0.0001)*	100 0 (*P* < 0.0001)*	96.6 3.4 (*P* = 0.0395)*	100 0 (*P* < 0.0001)*	100 0 (*P* < 0.0001)*	99.7 0.3 (*P* < 0.0001)*
**L221R** (NI)	T (**L**) G (**R**)	99.1 0.9	NI	NI	NI	NI	NI	NI
**C224Y** (rs772893975)	G (**C**) A (**Y**)	97.9 2.1	ND	ND	ND	ND	ND	100 0 (*P* < 0.0001)*
**T238A** (rs201709403)	A (**T**) G (**A**)	95.8 4.2	100 0 (*P* < 0.0001)*	100 0 (*P* < 0.0001)*	100 0 (*P* < 0.0001)*	100 0 (*P* < 0.0001)*	100 0 (*P* < 0.0001)*	100 0 (*P* < 0.0001)*
**T238I (NI)**	C (**T**) T (**I)**	97.6 2.4	NI	NI	NI	NI	NI	NI
**R248K** (rs61748819)	G (**R**) A (**K**)	89.0 11.0	100 0 (*P* < 0.0001)*	100 0 (*P* < 0.0001)*	89.0 11.0 (*P* = 1.0)	99.0 1.0 (*P* < 0.0001)*	100 0 (*P* < 0.0001)*	99.0 1.0 (*P* < 0.0001)*
**C320Y** (rs61999342)	G (**C**) A (**Y**)	99.7 0.3	ND	ND	ND	ND	ND	100 0 (*P* < 0.0001)*
APOBEC3D synonymous allele frequencies (%)								
**L221 L** (rs769426665)	G (**L**) C (**L**)	99.10.9	ND	ND	ND	ND	ND	100 0 (*P* < 0.0001)*
**T316 T** (rs184448269)	C (**T**) T (**T**)	97.9 2.1	100 0 (*P* < 0.0001)*	100 0 (*P* < 0.0001)*	98.8 1.2 (*P* = 0.2917)	99.6 0.4 (*P* = 0.0169)*	100 0 (*P* < 0.0001)*	99.9 0.1 (*P* < 0.0001)*
APOBEC 3F nonsynonymous allele frequencies (%)								
**R48P** (rs35053197)	G (**R**) C (**P**)	96.1 3.9	100 0 (*P* < 0.0001)*	100 0 (*P* < 0.0001)*	96.9 3.1 (*P* = 0.4762)	99.4 0.6 (*P* = 0.0003)*	99.4 0.6 (*P* = 0.0001)*	99.7 0.3 (*P* < 0.0001)*
**A78V** (rs5750728)	C (**A**) T (**V**)	79.9 20.1	29 71 (*P* < 0.0001)*	51 49 (*P* < 0.0001)*	80 20 (*P* = 0.9371)	38 62 (*P* < 0.0001)*	39 61 (*P* < 0.0001)*	52.3 47.8 (*P* < 0.0001)*
**I87L** (rs146543452)	A (**I**) C (**L**)	99.7 0.3	100 0 (*P* = 0.2340)	100 0 (*P* = 0.2344)	99.0 1.0 (*P* = 0.4897)	100 0 (*P* = 0.3074)	100 0 (*P* = 0.2395)	100 0 (*P* < 0.0001)*
**Q87L** (rs114704208)	A (**Q**) T (**L**)	97.1 2.9	100 0 (*P* < 0.0001)*	100 0 (*P* < 0.0001)*	94.5 5.5 (*P* = 0.0606)	99.6 0.4 (*P* = 0.0019)*	100 0 (*P* < 0.0001)*	99.7 0.3 (*P* < 0.0001)*
**A108S** (rs2020390)	G (**A**) T (**S**)	62.3 37.7	29.0 71.0 (*P* < 0.0001)*	51.0 49.0 (*P* = 0.0005)*	68.0 32.0 (*P* = 0.0600)	37.0 63.0 (*P* < 0.0001)*	40.0 60.0 (*P* = 0.0001)*	52.4 47.6 (*P* < 0.0001)*
**V231I** (rs2076101)	G (**V**) A (**I**)	89.0 11.0	29.0 71.0 (*P* < 0.0001)*	51.0 49.0 (*P* < 0.0001)*	81.0 19.0 (*P* = 0.0008)*	38.0 62.0 (*P* < 0.0001)*	39.0 61.0 (*P* < 0.0001)*	48.4 51.6 (*P* < 0.0001)*
**Y307C** (rs12157816)	A (**Y**) G (**C**)	95.1 4.9	100 0 (*P* < 0.0001)*	98.0 2.0 (*P* = 0.0133)*	97.0 3.0 (*P* = 0.1148)	98.0 2.0 (*P* = 0.0225)*	100 0 (*P* < 0.0001)*	98.7 1.3 (*P* < 0.0001)*
APOBEC3F synonymous allele frequencies (%)								
**I117I** (NI)	C (**I**) T (**I**)	99.4 0.6	NI	NI	NI	NI	NI	NI
**S118S** (rs35928287)	C (**S**) T (**S**)	86.7 13.3	ND	ND	ND	ND	ND	99.7 0.3 (*P* < 0.0001)*
**R143R** (rs4821862)	C (**R**) T (**R**)	41.958.1	29.0 71.0 (*P* < 0.0001)*	51.0 49.0 (*P* = 0.0040)*	45.0 55.0 (*P* = 0.2781)	36.0 64.0 (*P* = 0.1038)	39.0 61.0 (*P* = 0.4613)	45.5 54.6 (*P* = 0.1787)
**Y196Y** (rs765418322)	T (**Y**) C (**Y**)	89.6 10.4	ND	ND	ND	ND	ND	100 0 (*P* < 0.0001)*
**S229S** (rs549550231)	A (**S**) G (**S**)	99.4 0.6	ND	ND	ND	ND	ND	100 0 (*P* < 0.0001)*
**E245E** (rs113109079)	G (**E**) A (**E**)	97.1 3.9	100 0 (*P* < 0.0001)*	100 0 (*P* < 0.0001)*	99.0 1.0 (*P* = 0.0231)*	100 0 (*P* < 0.0001)*	100 0 (*P* < 0.0001)*	100 0 (*P* < 0.0001)*
**S327S** (rs35895636)	C (**S**) T (**S**)	90.6 9.4	100 0 (*P* < 0.0001)*	100 0 (*P* < 0.0001)*	98.3 1.7 (*P* < 0.0001)*	100 0 (*P* < 0.0001)*	100 0 (*P* < 0.0001)*	99.8 0.2 (*P* < 0.0001)*
APOBEC 3G nonsynonymous allele frequencies (%)								
**H186R** (rs8177832)	A (**H**) G (**R**)	63.0 37.0	92.8 7.2 (*P* < 0.0001)*	97.0 3.0 (*P* < 0.0001)*	57.0 43.0 (*P* = 0.0530)	92.5 7.5 (*P* < 0.0001)*	99.2 0.8 (*P* < 0.0001)	93.6 6.4 (*P* < 0.0001)*
**R256H** (rs17000736)	G (**R**) A (**H**)	98.8 1.2	100 0 (*P* = 0.0036)*	100 0 (*P* = 0.0037)*	98.6 1.4 (P = 1.0)	100 0 (*P* = 0.0107)*	100 0 (*P* = 0.0040)8	100 0 (*P* = 0.0002)*
**Q275E** (rs17496046)	C (**Q**) G (**E**)	82.1 17.9	97.3 2.7 (*P* < 0.0001)*	94.6 5.4 (*P* < 0.0001)*	87.5 12.5 (*P* = 0.0119)*	96.0 4.0 (*P* < 0.0001)*	98.7 1.3 (*P* < 0.0001)*	94.3 5.7 (*P* < 0.0001)*
**G363R** (rs148267053)	G (**G**) A (**R**)	94.8 5.2	100 0 (*P* < 0.0001)*	100 0 (*P* < 0.0001)*	98.6 1.4 (*P* = 0.3220)	99.9 0.1 (*P* = 0.0017)*	100 0 (*P* < 0.0001)*	100 0 (*P* < 0.0001)*
APOBEC3G synonymous allele frequencies (%)								
**S60S** (rs112603901)	C (**S**) T (**S**)	94.5 5.5	100 0 (*P* < 0.0001)*	100 0 (*P* < 0.0001)*	99.7 0.3 (*P* < 0.0001)*	100 0 (*P* < 0.0001)*	100 0 (*P* < 0.0001)*	100 0 (*P* < 0.0001)*
**A109A** (rs375760983)	C (**A**) T (**A**)	99.7 0.3	ND	ND	ND	ND	ND	100 0 (*P* < 0.0001)*
**F119F** (rs5757465)	T (**F**) C (**F**)	99.7 0.3	77.6 22.4 (*P* < 0.0001)*	55.3 44.7 (*P* < 0.0001)*	97.1 2.9 (*P* = 0.0035)*	60.2 39.8(*P* < 0.0001)*	55.5 44.5 (*P* < 0.0001)*	62.8 37.2 (*P* < 0.0001)*
**L371 L** (rs11545130)	C (**L**) T (**L**)	97.9 2.1	100 0 (*P* < 0.0001)*	100 0 (*P* < 0.0001)*	97.0 3.0 (*P* = 0.4614)	99.6 0.4 (*P* = 0.0157)*	100 0 (*P* < 0.0001)*	99.7 0.3 (*P* < 0.0001)*
APOBEC 3H nonsynonymous allele frequencies (%)								
**N15Δ** (rs140936762)	CAA(**N**) Δ	40.2 59.8	74 26 (*P* < 0.0001)*	66 34 (*P* < 0.0001)*	69 31 (*P* < 0.0001)*	72 28 (*P* < 0.0001)*	60 40 (*P* < 0.0001)*	65.7 34.3 (*P* < 0.0001)*
**R18L** (rs139293)	G (**R**) T (**L**)	89.8 10.2	84.1 15.9 (*P* = 0.0192)*	70.7 29.3 (*P* < 0.0001)*	93.0 7.0 (*P* = 0.0972)	75.8 24.2 (*P* < 0.0001)*	69.4 30.6 (*P* < 0.0001)*	73.2 26.8 (*P* < 0.0001)*
**G105R** (rs139297)	G (**G**) C (**R**)	2.2 97.8	68.5 31.5 (*P* < 0.0001)*	53.6 46.4 (*P* < 0.0001)*	12.5 87.5 (*P* < 0.0001)*	61.7 38.3 (*P* < 0.0001)*	57.1 42.9 (*P* < 0.0001)*	51.9 48.1 (*P* < 0.0001)*
**K121E** (rs139298)	A (**K**) G (**E**)	3.0 97.0	68.5 31.5 (*P* < 0.0001)*	52.3 47.7 (*P* < 0.0001)*	12.5 87.5 (*P* < 0.0001)*	61.2 38.8 (*P* < 0.0001)*	56.1 43.9 (*P* < 0.0001)*	51.5 48.5 (*P* < 0.0001)*
**K140E** (rs139300)	A (**K**) G (**E**)	0100	0100 (*P* = 1.0)	0100 (*P* = 1.0)	0100 (*P* = 1.0)	0100 (*P* = 1.0)	0100 (*P* = 1.0)	0100 (*P* = 1.0)
**E178D** (rs139302)	G (**E**) C (**D**)	6.4 93.6	67.0 33.0 (*P* < 0.0001)*	54.6 45.4 (*P* < 0.0001)*	15.3 84.7 (*P* < 0.0001)*	65.4 34.6 (*P* < 0.0001)*	56.1 43.9 (*P* < 0.0001)*	52.3 47.7 (*P* < 0.0001)*
APOBEC3H synonymous allele frequencies (%)								
**T43 T** (rs139294)	G (**T**) C (**T**)	7.1 92.9	66.7 33.3 (*P* < 0.0001)*	54.6 45.4 (*P* < 0.0001)*	17.2 82.8 (*P* < 0.0001)*	62.0 38.0 (*P* < 0.0001)*	56.0 44.0 (*P* < 0.0001)*	52.4 47.6 (*P* < 0.0001)*

Notes:

NI = Not Identified previously

ND = Not Determined in 1000 genomes database

* = Significant (*P* value ≤0.05) Fisher’ s Exact Test used for EAS, EUR, AFR, AMR, SAS

Chi Squared Test used for ExAC

In a previous study by Duggal and colleagues that compared Apobec 3 variation between Africans, Asian and Europeans, nonsynonymous variation in A3D (R97C, R248K); A3F (A108S, V231I, Y307C); A3G (H186R, E275Q (now Q275E) and A3H (15Δ, R18L, R105G (now G105R), E121K/D, E178D) were reported [13]. Our data suggest that several variants occur more frequently in our South African population than in the “African” population they previously studied [13]. These include R97C and T238A in A3D; A108S and Y307C in A3F; Q275E in A3G and N15**Δ,** R18L, G105R and E178D in A3H (Table 4).

Overall, the EAS, EUR, AMR, SAS populations and the ExAC consortium database showed a higher level of Apobec 3 conservation than our study population (Table 4). For example, the A3D sequences in these populations were more closely related to the reference GRCh37 human genome (98–100%) than in our SA population, resulting in signficant *p*-values for all the variant comparisons where allele frequencies were available. In the case of A3F and A3G, several variants were also present more frequently than in the other populations (see Table 4). In the case of A3H, the N15**Δ** variant was clearly present in significantly higher frequency in our population compared to the others. This was also the case for all of the other observed variants, with the exception of R18L and K140E, which as discussed above is likely a sequencing error or an extremely rare variant. R18L was significantly lower in all of the populations, with the exception of the AFR population, where it was not significantly different. This is in contrast to all of the other variants, which were significantly higher in our SA population than in the AFR population. In the case of A3 D, F and G, the frequency for some of the variants were also significantly higher in our population than in the AFR population, whereas others showed more similar allele frequencies (see Table 4).

The term “Africans” has been loosely used to describe datasets generated from different parts of the African continent. To provide a more accurate comparison, we next compared the variants detected in our study to the various components of the AFR data set that consist of more specific African subpopulations or people of African descent (Table 5). These included Americans of African Ancestry in USA (ASW); African Caribbeans in Barbados (ACB); Gambians in the Western Gambia (GWD); Esan in Nigeria (ESN); Luhya in Webuye, Kenya (LWK); Mende in Sierra Leone (MSL) and Yoruba in Ibadan, Nigeria (YRI). We noticed higher levels of single nucleotide changes in our population (with significant *p*-values) compared to most of the other populations for the following variants: T238A in A3D, S327S in A3F, S60S, Q275E and G363R in A3G and all of the variants in A3H with the exception of R18L (and K140E-see above). (Table 5). Notably, the variant frequency of R97C in A3D is almost the same as in ASW and LWK but higher than in the other populations. The frequency of R48P in A3F and the frequency of R256H in A3G were similar among all Africans.

**Table 5:** Comparison of A3D, A3F, A3G and A3H allele frequencies between our South African population (**SA**) and other African populations in the 1000 Genome Project including: the African Caribbeans in Barbados (**ACB**), Americans of African Ancestry in USA (**ASW**), Esan in Nigeria (**ESN**), Gambian in the Western Gambia (**GWD**), Luhya in Webuye, Kenya (**LWK**), Mende in Sierra Leone (**MSL**) and Yoruba in Ibadan, Nigeria (**YRI**)

Amino acid change and variant ID	Allele (2n)	SA (336)	ACB (192)	ASW (122)	ESN (198)	GWD (226)	LWK (198)	MSL (170)	YRI (216)
APOBEC 3D nonsynonymous allele frequencies (%)									
**R6K (**NI)	G (**R**) A (**K**)	94.9 5.1	NI	NI	NI	NI	NI	NI	NI
**R97C** (rs75858538)	C (**R**) T (**C**)	93.8 6.2	97 3 (*P* = 0.1506)	94 6 (*P* = 1.0)	97 3 (*P* = 0.0614)	98 2 (*P* = 0.0253)*	94 6 (*P* = 1.0)	98 2 (*P* = 0.0262)*	97 3 (*P* = 0.1631)
**L221R** (NI)	T (**L**) G (**R**)	99.1 0.9	NI	NI	NI	NI	NI	NI	NI
**C224Y** (rs772893975)	G (**C**) A (**Y**)	97.9 2.1	ND	ND	ND	ND	ND	ND	ND
**T238A** (rs201709403)	A (**T**) G (**A**)	95.8 4.2	100 0 (*P* = 0.0030)*	99 1 (*P* = 0.1322)	100 0 (*P* = 0.0016)*	100 0 (*P* = 0.0012)*	100 0 (*P* = 0.0016)*	100 0 (*P* = 0.0036)*	100 0 (*P* = 0.0013)*
**T238I** (NI)	C (**T**) T (**I)**	97.6 2.4	NI	NI	NI	NI	NI	NI	NI
**R248K** (rs61748819)	G (**R**) A (**K**)	89.0 11.0	91 9 (*P* = 0.4596)	96 4 (*P* = 0.0267)*	86 14 (*P* = 0.3375)	92 8 (*P* = 0.3890)	87 13 (*P* = 0.5785)	86 14 (*P* = 0.4668)	87 13 (*P* = 0.5018)
**C320Y** (rs61999542)	G (**C**) A (**Y**)	99.7 0.3	ND	ND	ND	ND	ND	ND	ND
APOBEC3D synonymous allele frequencies (%)									
**L221 L** (rs769426665)	G (**L**) C (**L**)	99.1 0.9	ND	ND	ND	ND	ND	ND	ND
**T316 T** (rs184448269)	C (**T**) T (**T**)	97.9 2.1	99 1 (*P* = 0.3657)	99 1 (*P* = 0.3657)	98 2 (*P* = 0.3657)	99 1 (*P* = 0.3657)	99 1 (*P* = 0.3657)	98 2 (*P* = 0.3657)	99 1 (*P* = 0.3657)
APOBEC 3F nonsynonymous allele frequencies (%)									
**R48P** (rs35053197)	G (**R**) C (**P**)	96.1 3.9	98 2 (*P* = 0.3074)	98 2 (*P* = 0.3670)	95 5 (*P* = 0.8202)	99 1 (*P* = 0.1100)	95 5 (*P* = 0.6559)	98 2 (*P* = 0.2763)	95 5 (*P* = 0.6663)
**A78V** (rs5750728)	C (**A**) T (**V**)	79.9 20.1	78 22 (*P* = 0.6518)	73 27 (*P* = 0.1236)	82 18 (*P* = 0.6453)	77 23 (*P* = 0.3943)	76 24 (0.3756)	85 15 (*P* = 0.2183)	86 14 (*P* = 0.0798)
**I87L** (rs146543452)	A (**I**) C (**L**)	99.7 0.3	100 0 (*P* = 1)	100 0 (*P* = 1)	100 0 (*P* = 1)	98 2 (*P* = 0.0878)	99 1 (*P* = 1)	99 1 (*P* = 1)	100 0 (*P* = 1)
**Q87L** (rs114704208)	A (**Q**) T (**L**)	97.1 2.9	92.2 7.8 (*P* = 0.0171)*	93.4 6.6 (*P* = 0.0994)	96.5 3.5 (*P* = 0.7961)	96.0 4.0 (*P* = 0.6287)	93.0 7.0 (*P* = 0.0718)	94.7 5.3 (*P* = 2141)	94.4 5.6 (*P* = 0.1738)
**A108S** (rs2020390)	G (**A**) T (**S**)	62.3 37.7	67 33 (*P* = 0.2922)	66 34 (*P* = 0.5796)	69 31 (*P* = 0.1268)	62 38 (*P* = 0.8572)	65 35 (*P* = 0.6371)	76 24 (*P* = 0.0031)*	70 30 (*P* = 0.0619)
**V231I** (rs2076101)	G (**V**) A (**I**)	89.0 11.0	79 21 (*P* = 0.0041)*	73 27 (*P* = 0.0001)*	84 16 (*P* = 0.1053)	78 22 (*P* = 0.0007)*	80 20 (*P* = 0.0092)*	85 15 (*P* = 0.2485)	87 13 (*P* = 0.5829)
**Y307C** (rs12157816)	A (**Y**) G (**C**)	95.1 4.9	95 5 (*P* = 0.8368)	98 2 (*P* = 0.1703)	96 4 (*P* = 0.5129)	98 2 (*P* = 0.1649)	98 2 (*P* = 0.1489)	96 4 (*P* = 0.8218)	95 5(*P* = 1.0)
APOBEC3F synonymous allele frequencies (%)									
**I117I** (NI)	C (**I**) T (**I**)	99.4 0.6	NI	NI	NI	NI	NI	NI	NI
**S118S** (rs35928287)	C (**S**) T (**S**)	86.7 13.3	ND	ND	ND	ND	ND	ND	ND
**R143R** (rs4821862)	C (**R**) T (**R**)	41.9 58.1	45 55 (*P* = 0.5775)	45 55 (*P* = 5892)	39 61 (*P* = 0.6433)	50 50 (*P* = 0.0794)	45 55 (*P* = 0.4624)	46 54 (*P* = 0.3372)	47 53 (*P* = 0.2840)
**Y196Y** (rs765418322)	T (**Y**) C (**Y**)	89.6 10.4	ND	ND	ND	ND	ND	ND	ND
**S229S** (rs549550231)	A (**S**) G (**S**)	99.4 0.6	ND	ND	ND	ND	ND	ND	ND
**E245E** (rs113109079)	G (**E**) A (**E**)	97.1 2.9	99 1 (*P* = 0.2175)	98 2 (*P* = 0.7356)	100 0 (*P* = 0.0139)*	98 2 (*P* = 0.5717)	99 1 (*P* = 0.0971)	98 2 (*P* = 0.5517)	98 2 (*P* = 0.1350)
**S327S** (rs35895636)	C (**S**) T (**S**)	90.6 9.4	98 2 (*P* = 0.0013)*	99 1 (*P* = 0.0006)*	96 4 (*P* = 0.0126)*	100 0 (*P* = 0.0001)*	97 3 (*P* = 0.0064)*	100 0 (*P* = 0.0001)*	98 2 (*P* = 0.0004)*
APOBEC 3G nonsynonymous allele frequencies (%)									
**H186R** (rs8177832)	A (**H**) G (**R**)	63.037.0	56 44 (*P* = 0.1147)	75 25 (*P* = 0.0249)*	49 51 (*P* = 0.0026)*	57 43 (*P* = 0.1344)	68 32 (*P* = 0.3013)	49 51 (*P* = 0.0030)*	52 48 (*P* = 0.0101)*
**R256H** (rs17000736)	G (**R**) A (**H**)	98.8 1.2	98 2 (*P* = 0.7118)	98 2 (*P* = 0.6631)	99 1 (*P* = 0.6550)	97 3 (*P* = 0.1316)	98 2 (*P* = 1.0)	99 1 (*P* = 0.6660)	99 1 (*P* = 1.0)
**Q275E** (rs17496046)	C (**Q**) G (**E**)	82.117.9	90 10 (*P* = 0.0026)*	91 9 (*P* = 0.0064)*	86 14 (*P* = 0.0725)	87 13 (*P* = 0.0482)*	83 17 (*P* = 0.3526)	91 9 (*P* = 0.0027)*	87 13 (*P* = 0.0473)*
**G363R** (rs148267053)	G (**G**) A (**R**)	94.8 5.2	98 2 (*P* = 0.1066)	99 1(*P* = 0.0532)	100 0 (*P* = 0.0005)*	98 2(*P* = 0.0429)*	99 1(*P* = 0.0142)*	98 2 (*P* = 0.0902)	98 2 (*P* = 0.1209)
APOBEC3G synonymous allele frequencies (%)									
**S60S** (rs112603901)	C (**S**) T (**S**)	94.5 5.5	99 1 (*P* = 0.0027)*	98 2(*P* = 0.1191)	100 0 (*P* = 0.0002)*	100 0 (*P* = 0.0001)*	99 1 (*P* = 0.0026)*	100 0 (*P* = 0.0006)*	100 0 (*P* = 0.0001)*
**A109A** (rs375760983)	C (**A**) T (**A**)	99.7 0.3	ND	ND	ND	ND	ND	ND	ND
**F119F** (rs5757465)	T (**F**) C (**F**)	99.7 0.3	93 7 (*P* = 0.0001)*	89 11 (*P* = 0.0001)*	100 0(*P* = 1.0)	98 2 (*P* = 0.1639)	98 2 (*P* = 0.1507)	99 1 (*P* = 1.0)	99 1 (*P* = 0.5654)
**L371 L** (rs11545130)	C (**L**) T (**L**)	97.9 2.1	98 2 (*P* = 0.7523)	98 2 (*P* = 1.0)	97 3 (*P* = 0.7698)	98 2 (*P* = 1.0)	95 5 (*P* = 0.1239)	98 2 (*P* = 1.0)	95 5 (*P* = 0.1299)
APOBEC 3H nonsynonymous allele frequencies (%)									
**N15Δ** (rs140936762)	CAA(**N**) Δ	40.2 59.8	71 29 (*P* < 0.0001)*	70 30 (*P* < 0.0001)*	61 39 (*P* = 0.0004)*	78 22 (*P* < 0.0001)*	62 38 (*P* = 0.0002)*	75 25 (*P* < 0.0001)*	68 32 (*P* < 0.0001)*
**R18L** (rs139293)	G (**R**) T (**L**)	89.8 10.2	93 7 (*P* = 0.3230)	87 13 (*P* = 0.3885)	93 7 (*P* = 0.3212)	94 6 (*P* = 0.0972)	94 6 (*P* = 0.0873)	93 7 (*P* = 0.3053)	96 4 (*P* = 0.0076)*
**G105R** (rs139297)	G (**G**) C (**R**)	2.2 97.8	15 85 (*P* < 0.0001)*	25 75 (*P* < 0.0001)*	10 90 (*P* = 0.0007)*	13 87 (*P* < 0.0001)*	9 91 (*P* = 0.0012)*	11 89 (*P* < 0.0002)*	9 91 (*P* < 0.0016)*
**K121E** (rs139298)	A (**K**) G (**E**)	3.0 97.0	15 85 (*P* < 0.0001)*	25 75 (*P* < 0.0001)*	10 90 (*P* = 0.0025)*	13 87 (*P* < 0.0001)*	9 91 (*P* = 0.0070)*	11 89 (*P* = 0.0008)*	9 91 (*P* = 0.0086)*
**K140E** (rs139300)	A (**K**) G (**E**)	0100	0100 (*P* = 1.0)	0100 (*P* = 1.0)	0100 (*P* = 1.0)	0100 (*P* = 1.0)	0100 (*P* = 1.0)	0100 (*P* = 1.0)	0100 (*P* = 1.0)
**E178D** (rs139302)	G (**E**) C (**D**)	6.4 93.6	17 83 (*P* = 0.0004)*	29 71 (*P* < 0.0001)*	11 89 (*P* = 0.0900)	16 84 (*P* = 0.0007)*	16 84 (*P* = 0.0018)*	13 87 (*P* = 0.0248)*	11 89 (*P* = 0.0991)
APOBEC3H synonymous allele frequencies (%)									
**T43 T** (rs139294)	G (**T**) C (**T**)	7.1 92.9	22 78 (*P* < 0.0001)*	30 70 (*P* < 0.0001)*	16 84 (*P* = 0.0026)*	16 84 (*P* = 0.0024)*	13 87 (*P* = 0.0545)	17 83 (*P* = 0.0016)*	13 87 (*P* = 0.0439)*

Note:

NI = Not Identified previously

ND = Not Determined in 1000 genomes database

*= Significant (*P* value ≤0.05); Fisher’ s Exact Test used

## Discussion

In this study, we characterized SNPs and indels within the coding exons of several human APOBEC3 genes (A3D, A3F, A3G and A3H) to document the level of diversity in these genes in HIV infected individuals in a diverse South African population residing in the Limpopo Province in Northern South Africa. We observed a high level of A3 diversity and a higher prevalence of certain variants than has previously been observed in other African populations. Interestingly, some of these variants have previously been linked to HIV disease progression [14, 39, 42] (see below). The use of next generation sequencing also allowed the identification of SNP genotypes that were not previously identified in South Africa, since previous studies used older methods such as TaqMan, SNP array genotyping assays, restriction fragment length polymorphism (RFLP) or Sanger sequencing [39].

Common variants in APOBEC3 genes have been intensively studied and many have been found to have differential effects on antiviral activity [7, 13, 14, 39, 42]. For example, the variants R97C and R248K in A3D have been reported to moderately decrease antiviral activity [13]. In contrast, the A3F variants A108S, V231I and Y307C have been reported to have potent antiviral activity against HIV-1 ΔVif strains [43, 44]. SNPs in A3G can also alter its antiviral activity and sometimes enhance the rate of HIV-1 disease progression, as reported in a cohort of HIV-1 subtype C infected South African women and a US based cohort of African Americans [14, 39]. In particular, the H186R variant has previously been associated with more rapid decline in CD4+ cells and accelerated disease progression [14, 39, 42]. Our study shows that this variant is present in much higher frequency in our SA population than in the non-African populations and in the ExAC database (Table 4). This variant is similar in prevalence in our population to that in several other African populations (Table 5).

Recent studies have shown A3H as the most polymorphic member of the A3 family. The A3H variants (15Δ, R18L, G105R, K121E, E178D), which make up 7 different haplotypes, have been functionally described in other studies, showing varying protein expression and stability [8, 11, 16, 45–48]. Data from the 1000 genome project suggest that stable A3H haplotypes (II, V and VII) predominate in Africa while unstable haplotypes (I, III, IV, VI) are more prevalent in Asia [15], Interestingly, the unstable A3H haplotypes III and IV (which cannot restrict HIV) were unexpectedly high among our study population. This can be attributed mainly to the high prevalence of the deletion at amino acid residue 15 (Tables 2, 3, 4 and 5) that showed an allele frequency of almost 60% in our population. This is very different from what was reported in previous studies of Africans, in which stable A3H haplotypes were reported to be dominant [15] (see also Table 5). Data from two recent studies illustrate that stable A3H haplotypes may function as contemporary HIV-1 restriction factors, contributing to limiting viral replication and rates of transmission [12, 15]. It is unclear what role, if any, the unstable A3H haplotype III and IV, which are the only ones present in over 40% of the patients we analyzed, may play in the high prevalence and transmission of HIV-1 in Limpopo.

Because HIV-1 Vif acts as an antagonist to APOBEC proteins including A3H, we speculate that the distribution of stable versus unstable A3H haplotypes in our study might also influence Vif variation in HIV in our study population. Studies performed in primary CD4+ lymphocytes have shown that HIV-1 Vif variants with certain amino acid residues (F39 and H48), known as hyper Vifs, are better capable of neutralizing stable A3H genotypes, implying that HIV-1 Vif might adapt to the A3H haplotype in a particular population [15]. We are presently analyzing HIV-1 Vif sequences from our study subjects in order to determine a possible correlation between the A3H haplotypes and HIV-1 Vif genetic variation in this rural area of South Africa.

All the subjects in this study were HIV infected and were mostly at the chronic stage of infection. Even though there is to date no strong evidence that APOBEC 3 genotypes significantly affect HIV infection risk, it remains possible that HIV-1 negative subjects in Limpopo would present a significant different A3 profile. If this turns out to be the case, it could imply that A3 genotypes either alone or in combination influence HIV transmission. It will thus be important to compare HIV positive and negative individuals in future studies of APOBEC3 variants in this region. It is also possible that the overall APOBEC3 expression landscape may turn out to affect disease progression. However, exploring this hypothesis would require studies in which clinical data are correlated with APOBEC 3 expression. Future studies of this kind are clearly warranted, since a previous report comparing HIV-1 non-controllers versus long-term non-progressors (LTNP) reported that LTNPs express higher levels of A3G and A3F proteins [49].

## Conclusions

We have shown that significant A3 variation exists among HIV patients in an ethnically diverse population in Northern South Africa, by providing extensive data for 4 different A3 genes that are known to restrict HIV infection, but have previously only been sparsely studied in African populations. Our NGS results provide a baseline for future studies that could functionally characterize the SNPs identified in the APOBEC3 genes in this population and specifically analyze how they affect restriction of HIV replication and Vif function. Such studies will serve to increase our understanding of how the APOBEC3 protein landscape might have shaped the HIV epidemic in Northern South Africa.

## Additional files

Additional file 1: **Table S1.** Study Participants Demographic Information: Gender, Age, Ethinicity, Geography, HIV Viral Load, CD4+ cell count, Apobec3 genes sequenced. (DOCX 178 kb)

Additional file 2: **Table S2.** Apobec 3D- Linkage Disequilibrium Calculations: D’ and R^2^ values. (PDF 42 kb)

Additional file 3: **Table S3.** Apobec 3F- Linkage Disequilibrium Calculations: D’ and R^2^ values. (PDF 52 kb)

Additional file 4: **Table S4.** Apobec 3G- Linkage Disequilibrium Calculations: D’ and R^2^ values. (PDF 33 kb)

Additional file 5: **Table S5.** Apobec 3H- Linkage Disequilibrium Calculations: D’ and R^2^ values. (PDF 50 kb)

## Abbreviations

1000G: 1000 genomes
A3D: APOBEC3D
A3F: APOBEC3F
A3G: APOBEC3G
A3H: APOBEC3H
ACB: African Caribbeans in Barbados African
AFR: African
AMR: Ad Mixed American
APOBEC3: Apolipoprotein B mRNA-editing enzyme, catalytic polypeptide-like 3
ASW: Americans of African Ancestry in USA
EAS: East Asian
ESN: Esan in Nigeria
EUR: European
ExAC: Exome Aggregation Consortium
GWD: Gambians in the Western Gambia
HWE: Hardy-Weinberg Equilibrium
LD: Pairwise linkage disequilibrium
LWK: Luhya in Webuye, Kenya
MSL: Mende in Sierra Leone
NGS: Next generation sequencing
PBMC: Peripheral blood mononuclear cells
PCR: Polymerase chain reaction
SAS: South Asian
SNP: Single nucleotide polymorphism
YRI: Yoruba in Ibadan, Nigeria
