# Supplementary material for: Characterization of APOBEC3 variation in a population of HIV-1 infected individuals in northern South Africa
Source: BMC Med Genet. 2019 Jan 19;20:21. doi: 10.1186/s12881-018-0740-4 (PMC6339282; doi:10.1186/s12881-018-0740-4)
Supplement: Supplementary file 1 — Table S1. Study Participants Demographic Information: Gender, Age, Ethinicity, Geography, HIV Viral Load, CD4+ cell count, Apobec3 genes sequenced. (DOCX 178 kb) [file 12881_2018_740_MOESM1_ESM.docx]

| **Patient ID** | **Gender** | **Age** | **Ethnicity** | **Geography (district)** | **HIV Viral Load (copies/ml)** | **CD4+ cell count (cells/μl)** | **APOBEC3 genes sequenced** |
| --- | --- | --- | --- | --- | --- | --- | --- |
| BB01 | Female | 40 | Swati | Waterberg | 2364 | 414 | A3G, A3H |
| BB02 | Male | 61 | Tswana | Waterberg | 3090 | 338 | A3D, A3F, A3G, A3H |
| BB03 | Female | 36 | Tswana | Waterberg | 25748 | 186 | A3D, A3F, A3G, A3H |
| BB04 | Female | 37 | Bapedi | Waterberg | 1670 | 209 | A3F, A3G, A3H |
| BB05 | Female | 36 | Tswana | Waterberg | 1830 | 209 | A3G, A3H |
| BB07 | Female | 42 | Bapedi | Waterberg | 1830 | 209 | A3F, A3G, A3H |
| BB08 | Female | 39 | Bapedi | Waterberg | 1200 | 387 | A3D, A3F, A3H |
| BB09 | Male | 10 | Tswana | Waterberg | 10163 | 562 | A3D, A3F, A3H |
| BB10 | Female | 33 | Bapedi | Waterberg | 1773 | 600 | A3D, A3F, A3G, A3H |
| BB11 | Female | 38 | Tswana | Waterberg | 272241 | 783 | A3D, A3F, A3G, A3H |
| BB12 | Female | 49 | Bapedi | Waterberg | 859 | 788 | A3D, A3F, A3G, A3H |
| BB13 | Male | 43 | Tswana | Waterberg | 61354 | 42 | A3, A3F, A3G, A3H |
| BB14 | Male | 11 | Tswana | Waterberg | 2800 | 701 | A3H |
| BB15 | Male | 5 | Tswana | Waterberg | 104087 | 716 | A3F, A3G, A3H |
| BB16 | Male | 14 | Tswana | Waterberg | 1000 | 720 | A3F, A3G, A3H |
| BB17 | Female | 56 | Tswana | Waterberg | 342 | 1196 | A3F, A3G, A3H |
| BB18 | Female | 13 | Bapedi | Waterberg | 27503 | 540 | A3H |
| BB19 | Female | 40 | Bapedi | Waterberg | 916 | 247 | A3F, A3G, A3H |
| BB20 | Male | 44 | Tsonga | Waterberg | 48423 | 245 | A3D, A3F, A3G, A3H |
| BB21 | Female | 36 | Bapedi | Waterberg | 127219 | 170 | A3D, A3F, A3G, A3H |
| BB22 | Female | 29 | Bapedi | Waterberg | 226674 | 244 | A3F, A3G, A3H |
| BB23 | Female | 49 | Bapedi | Waterberg | 281380 | 142 | A3F, A3G, A3H |
| BB24 | Female | 18 | Bapedi | Waterberg | 1000 | 360 | A3D, A3F, A3G, A3H |
| BB25 | Female | 19 | Tswana | Waterberg | 1000 | 6 | - |
| BB26 | Female | 8 | Bapedi | Waterberg | 1000 | 230 | A3D, A3F, A3G, A3H |
| BB27 | Male | 41 | Bapedi | Waterberg | 1000 | 200 | - |
| BB28 | Male | 51 | Bapedi | Waterberg | 1000 | 120 | A3F, A3G, A3H |
| BB29 | Male | 30 | Tswana | Waterberg | 1000 | 189 | A3D, A3F, A3H |
| BB30 | Female | 14 | Tswana | Waterberg | 1000 | 300 | A3D, A3G, A3H |
| BB31 | Female | 41 | Bapedi | Waterberg | 1000 | 120 | A3D, A3F, A3G, A3H |
| BB32 | Female | 48 | Tswana | Waterberg | 53727 | 479 | A3D, A3F, A3G, A3H |
| BB33 | Male | 37 | Tswana | Waterberg | No data | No data | A3D, A3F, A3H |
| BBS04 | Female | 16 | Bapedi | Waterberg | 2985 | 727 | A3D, A3F, A3G, A3H |
| BBS05 | Female | 6 | Bapedi | Waterberg | 362599 | 176 | A3D, A3F, A3G, A3H |
| DF01 | Male | 50 | Venda | Vhembe | 56 | 714 | - |
| DF02 | Male | 68 | Venda | Vhembe | No data | No data | A3D, A3F, A3G, A3H |
| DF03 | Female | 64 | Venda | Vhembe | 1645 | 731 | - |
| DF04 | Female | 67 | Venda | Vhembe | <20 | 493 | A3D, A3F, A3G, A3H |
| DF05 | Female | 6 | Venda | Vhembe | 1346 | 1353 | A3D, A3F, A3G A3H |
| DF06 | Female | 48 | Venda | Vhembe | <20 | 381 | A3D, A3F, A3G, A3H |
| DF07 | Female | 28 | Venda | Vhembe | No data | No data | A3D, A3F, A3G, A3H |
| DF08 | Female | 40 | Venda | Vhembe | <20 | 543 | A3D, A3F, A3G, A3H |
| DF09 | Male | 13 | Venda | Vhembe | 15336 | 261 | A3D, A3F, A3G, A3H |
| DF10 | Female | 35 | Venda | Vhembe | No data | 362 | A3D, A3F, A3G, A3H |
| DF100 | Female | 39 | Venda | Vhembe | <20 | 388 | A3D, A3F, A3G, A3H |
| DF101 | Female | 39 | Venda | Vhembe | 1000 | No data | A3D, A3F, A3G, A3H |
| DF102 | Female | 26 | Venda | Vhembe | 16533 | 37 | A3D, A3F, A3G, A3H |
| DF103 | Female | 34 | Venda | Vhembe | 29449 | 300 | A3D, A3F, A3G, A3H |
| DF104 | Male | 59 | Venda | Vhembe | No data | 37 | A3D, A3F, A3G, A3H |
| DF105 | Male | 13 | Venda | Vhembe | 251978 | 242 | A3D, A3F, A3G, A3H |
| DF106 | Female | 7 | Venda | Vhembe | 23967 | 370 | A3D, A3F, A3G, A3H |
| DF107 | Female | 57 | Venda | Vhembe | 72049 | 254 | A3D, A3F, A3G, A3H |
| DF108 | Female | 43 | Venda | Vhembe | 7582 | 239 | A3D, A3F, A3G, A3H |
| DF109 | Female | 41 | Venda | Vhembe | 1123 | 344 | A3D, A3F, A3G, A3H |
| DF11 | Female | 38 | Venda | Vhembe | 155 | 251 | A3D, A3F, A3G, A3H |
| DF110 | Female | 39 | Venda | Vhembe | 1500 | No data | A3D, A3F, A3G, A3H |
| DF111 | Female | 48 | Venda | Vhembe | 1000 | No data | A3D, A3F, A3G, A3H |
| DF12 | Female | 46 | Venda | Vhembe | <20 | 359 | A3D, A3F, A3G, A3H |
| DF13 | Female | 55 | Venda | Vhembe | No data | 329 | A3D, A3F, A3G, A3H |
| DF14 | Female | 48 | Venda | Vhembe | LDL | 155 | A3D, A3F, A3G, A3H |
| DF15 | Male | 39 | Venda | Vhembe | 376 | 97 | A3D, A3F, A3G, A3H |
| DF16 | Male | 38 | Venda | Vhembe | 14246 | 211 | A3D, A3F, A3G, A3H |
| DF17 | Male | 46 | Venda | Vhembe | <20 | 347 | A3D, A3F, A3G, A3H |
| DF18 | Male | 44 | Venda | Vhembe | <20 | 609 | A3D, A3F, A3G, A3H |
| DF19 | Male | 36 | Venda | Vhembe | No data | No data | A3D, A3F, A3G, A3H |
| DF20 | Male | 78 | Venda | Vhembe | <20 | 624 | A3D, A3F, A3G, A3H |
| DF21 | Male | 33 | Venda | Vhembe | 520 | 639 | A3D, A3F, A3G, A3H |
| DF22 | Male | 50 | Venda | Vhembe | 520 | 639 | A3D, A3F, A3G, A3H |
| DF23 | Male | 63 | Venda | Vhembe | 39 | 283 | A3D, A3F, A3G, A3H |
| DF24 | Female | 60 | Venda | Vhembe | No data | No data | A3D, A3F, A3G, A3H |
| DF25 | Female | 36 | Venda | Vhembe | <20 | 579 | A3D, A3F, A3G, A3H |
| DF26 | Male | 42 | Venda | Vhembe | 100 | 236 | A3D, A3F, A3G, A3H |
| DF27 | Female | 32 | Venda | Vhembe | 259 | 296 | A3D, A3F, A3G, A3H |
| DF28 | Male | 49 | Venda | Vhembe | 1000 | 140 | A3D, A3F, A3G, A3H |
| DF29 | Male | 72 | Venda | Vhembe | 481 | 1022 | A3D, A3F, A3G, A3H |
| DF30 | Male | 48 | Venda | Vhembe | <20 | 137 | A3D, A3F, A3G, A3H |
| DF31 | Female | 42 | Venda | Vhembe | 715 | 708 | A3D, A3F, A3G, A3H |
| DF32 | Female | 38 | Venda | Vhembe | No data | No data | - |
| DF33 | Male | 51 | Venda | Vhembe | 75 | 323 | A3D, A3F, A3G, A3H |
| DF34 | Male | 46 | Venda | Vhembe | 399 | 512 | A3D, A3F, A3G, A3H |
| DF35 | Female | 60 | Venda | Vhembe | <20 | 322 | - |
| DF36 | Female | 62 | Venda | Vhembe | <20 | 302 | A3D, A3F, A3G, A3H |
| DF37 | Female | 39 | Venda | Vhembe | <20 | 828 | A3D, A3F, A3G, A3H |
| DF38 | Female | 10 | Venda | Vhembe | <20 | 573 | A3D, A3F, A3G, A3H |
| DF39 | Female | 47 | Venda | Vhembe | No data | No data | A3D, A3F, A3G, A3H |
| DF40 | Male | 46 | Venda | Vhembe | No data | No data | A3D, A3F, A3G, A3H |
| DF41 | Female | 40 | Venda | Vhembe | No data | No data | A3D, A3F, A3G, A3H |
| DF42 | Female | 32 | Venda | Vhembe | No data | No data | A3D, A3F, A3G, A3H |
| DF43 | Female | 18 | Venda | Vhembe | <20 | 230 | A3D, A3F, A3G, A3H |
| DF44 | Female | 52 | Venda | Vhembe | No data | No data | A3D, A3F, A3G |
| DF45 | Female | 50 | Venda | Vhembe | No data | No data | A3D, A3F, A3G, A3H |
| DF46 | Male | 62 | Venda | Vhembe | 337170 | 197 | A3D, A3F, A3G, A3H |
| DF47 | Male | 54 | Venda | Vhembe | 1000 | No data | A3D, A3F, A3G, A3H |
| DF48 | Female | 42 | Venda | Vhembe | 142222 | 141 | A3D, A3F, A3G |
| DF49 | Female | 8 | Venda | Vhembe | No data | No data | A3D, A3F, A3G, A3H |
| DF50 | Female | 36 | Venda | Vhembe | 3827 | 290 | A3D, A3F, A3G, A3H |
| DF51 | Female | 11 | Venda | Vhembe | 546710 | 151 | A3D, A3F, A3G, A3H |
| DF52 | Female | 20 | Venda | Vhembe | No data | No data | A3D, A3F, A3G, A3H |
| DF53 | Female | 31 | Venda | Vhembe | No data | No data | A3D, A3F, A3G, A3H |
| DF54 | Female | 60 | Venda | Vhembe | 2829 | 399 | A3D, A3F, A3G, A3H |
| DF55 | Male | 42 | Venda | Vhembe | 10212 | 424 | A3D, A3F, A3G, A3H |
| DF56 | Male | 10 | Venda | Vhembe | 10212 | 424 | A3D, A3F, A3G, A3H |
| DF57 | Female | 62 | Venda | Vhembe | No data | No data | A3D, A3F, A3G, A3H |
| DF58 | Male | 13 | Venda | Vhembe | No data | No data | A3D, A3F, A3G, A3H |
| DF59 | Female | 45 | Venda | Vhembe | No data | No data | A3D, A3F, A3G, A3H |
| DF60 | Female | 34 | Venda | Vhembe | 8262 | 212 | A3D, A3F, A3G, A3H |
| DF61 | Female | 37 | Venda | Vhembe | 1265 | 429 | A3D, A3F, A3G, A3H |
| DF62 | Female | 45 | Venda | Vhembe | 18344 | 135 | A3D, A3F, A3G, A3H |
| DF63 | Female | 11 | Venda | Vhembe | 3539 | 1005 | A3F, A3G, A3H |
| DF64 | Female | 5 | Venda | Vhembe | 10163 | 429 | A3D, A3F, A3G, A3H |
| DF65 | Female | 35 | Venda | Vhembe | 1230 | 331 | A3D, A3F, A3G, A3H |
| DF66 | Female | 33 | Venda | Vhembe | 50087 | 18 | A3D, A3F, A3G, A3H |
| DF67 | Male | 62 | Venda | Vhembe | 3760 | 125 | A3D, A3F, A3G, A3H |
| DF68 | Female | 34 | Venda | Vhembe | 8709 | 483 | A3D, A3F, A3G, A3H |
| DF69 | Female | 37 | Venda | Vhembe | 4640 | 178 | A3D, A3F, A3G, A3H |
| DF70 | Male | 14 | Venda | Vhembe | 18414 | 392 | A3F, A3G, A3H |
| DF71 | Male | 19 | Venda | Vhembe | 189711 | 292 | A3D, A3F, A3G, A3H |
| DF72 | Female | 62 | Venda | Vhembe | 16825 | 373 | A3D, A3F, A3G, A3H |
| DF73 | Male | 51 | Venda | Vhembe | 73389 | 432 | A3D, A3F, A3G, A3H |
| DF74 | Male | 13 | Venda | Vhembe | 28668 | 266 | A3D, A3F, A3G, A3H |
| DF75 | Male | 17 | Venda | Vhembe | 15520 | 240 | A3D, A3F, A3G, A3H |
| DF76 | Male | 15 | Venda | Vhembe | 26664 | 32 | A3D, A3F, A3G, A3H |
| DF77 | Female | 16 | Venda | Vhembe | 623250 | 246 | A3D, A3F, A3G, A3H |
| DF78 | Female | 30 | Venda | Vhembe | 11167 | 698 | A3D, A3F, A3H |
| DF79 | Female | 42 | Venda | Vhembe | 34894 | 296 | A3D, A3F, A3G, A3H |
| DF80 | Female | 27 | Venda | Vhembe | 13377 | 512 | A3D, A3F, A3G, A3H |
| DF81 | Male | 11 | Venda | Vhembe | 40565 | 264 | A3D, A3F, A3G, A3H |
| DF82 | Female | 53 | Venda | Vhembe | 2345 | 402 | A3D, A3F, A3G, A3H |
| DF83 | Male | 13 | Venda | Vhembe | 8191 | 417 | A3D, A3F, A3G, A3H |
| DF84 | Male | 18 | Venda | Vhembe | 15505 | 23 | A3D, A3F, A3G, A3H |
| DF85 | Female | 37 | Venda | Vhembe | 546710 | 215 | A3D, A3F, A3G, A3H |
| DF86 | Female | 52 | Venda | Vhembe | 5061 | 230 | A3D, A3G, A3H |
| DF87 | Male | 35 | Venda | Vhembe | 7109 | 557 | A3D, A3F, A3G, A3H |
| DF88 | Male | 8 | Venda | Vhembe | No data | No data | A3D, A3F, A3G, A3H |
| DF89 | Female | 42 | Venda | Vhembe | 1118 | 557 | A3D, A3F, A3G, A3H |
| DF90 | Male | 39 | Venda | Vhembe | 159436 | 5 | A3D, A3F, A3G, A3H |
| DF91 | Male | 46 | Venda | Vhembe | 23371 | 243 | A3D, A3F, A3G, A3H |
| DF92 | Male | 46 | Venda | Vhembe | 66307 | 2130 | A3D, A3F, A3G, A3H |
| DF94 | Male | 53 | Venda | Vhembe | 1030 | 347 | A3D, A3F, A3G, A3H |
| DF95 | Male | 42 | Venda | Vhembe | 7582 | 239 | A3D, A3F, A3G, A3H |
| DF96 | Male | 33 | Venda | Vhembe | 29067 | 933 | A3D, A3F, A3G, A3H |
| DF97 | Male | 53 | Venda | Vhembe | <20 | 623 | A3D, A3F, A3G, A3H |
| DF98 | Male | 4 | Venda | Vhembe | 1030 | 347 | A3D, A3F, A3G, A3H |
| DF99 | Male | 49 | Venda | Vhembe | No data | 464 | - |
| LP1111 | Female | 43 | Venda | Vhembe | 3785 | 365 | A3D, A3F, A3G, A3H |
| LP1112 | Male | 29 | Venda | Vhembe | 5890 | 289 | A3D, A3F, A3G, A3H |
| LP1119 | Female | 34 | Venda | Vhembe | 40 | 41 | A3D, A3F, A3G, A3H |
| LP1120 | Female | 39 | Venda | Vhembe | 340 | 50 | A3D, A3F, A3G, A3H |
| LP1121 | Female | 41 | Venda | Vhembe | 210 | 100 | A3D, A3F, A3G, A3H |
| LP1122 | Female | 38 | Venda | Vhembe | 32 | 57 | A3D, A3F, A3G, A3H |
| LP1123 | Female | 37 | Venda | Vhembe | 110 | 60 | A3D, A3F, A3G, A3H |
| LP1125 | Male | 34 | Venda | Vhembe | 37 | 64 | A3D, A3F, A3G, A3H |
| LP1126 | Male | 49 | Venda | Vhembe | 50 | 67 | A3D, A3F, A3G, A3H |
| LP1129 | Male | 50 | Venda | Vhembe | 97 | 41 | A3D, A3F, A3G, A3H |
| LP1131 | Male | 50 | Venda | Vhembe | 123 | 80 | A3D, A3F, A3G, A3H |
| LP1135 | Female | 57 | Venda | Vhembe | 31 | 85 | A3D, A3F, A3G, A3H |
| LP1136 | Female | 56 | Venda | Vhembe | 35 | 48 | A3D, A3F, A3G, A3H |
| LP1137 | Female | 64 | Venda | Vhembe | 106 | 89 | A3D, A3F, A3G, A3H |
| LP1138 | Female | 54 | Venda | Vhembe | 76 | 44 | A3D, A3F, A3G, A3H |
| LP1142 | Female | 55 | Venda | Vhembe | 300 | 95 | A3D, A3F, A3G, A3H |
| LP1148 | Female | 50 | Venda | Vhembe | 971 | 97 | A3D, A3F, A3G, A3H |
| LP1149 | Female | 56 | Venda | Vhembe | 100 | 38 | - |
| LP1150 | Female | 63 | Venda | Vhembe | 861 | 44 | A3D, A3F, A3G, A3H |
| LP1151 | Female | 55 | Venda | Vhembe | 521 | 46 | A3D, A3F, A3G, A3H |
| LP1152 | Male | 60 | Venda | Vhembe | 32 | 130 | A3D, A3F, A3G, A3H |
| LP1153 | Male | 58 | Venda | Vhembe | 585 | 47 | A3D, A3F, A3G, A3H |
| LP1154 | Female | 57 | Venda | Vhembe | 421 | 41 | A3D, A3F, A3G, A3H |
| LP1157 | Female | 55 | Venda | Vhembe | 824 | 48 | A3D, A3F, A3G, A3H |
| LP1158 | Male | 41 | Venda | Vhembe | 137 | 35 | - |
| LP1159 | Female | 60 | Venda | Vhembe | 944 | 155 | A3D, A3F, A3G, A3H |
| LP1160 | Male | 48 | Venda | Vhembe | 36 | 45 | A3D, A3F, A3G, A3H |
| LP1162 | Female | 58 | Venda | Vhembe | 677 | 110 | A3D, A3F, A3G, A3H |
| LP1163 | Female | 51 | Venda | Vhembe | 114 | 570 | - |
| LP1164 | Female | 55 | Venda | Vhembe | 522 | 149 | A3D, A3F, A3G, A3H |
| LP1165 | Male | 58 | Venda | Vhembe | 149 | 522 |  |
| LP1166 | Male | 48 | Venda | Vhembe | 34 | 42 | A3D, A3F, A3G, A3H |
| LP1167 | Male | 43 | Venda | Vhembe | 7639 | 140 | - |
| LP1169 | Male | 68 | Venda | Vhembe | 121000 | 42 | - |
| LP1170 | Female | 51 | Venda | Vhembe | 439 | 160 | A3D, A3F, A3G, A3H |
| LP1171 | Female | 56 | Venda | Vhembe | 5098 | 181 | A3D, A3F, A3G, A3H |
| LP1186 | Male | 15 | Venda | Vhembe | 33 | 129 | A3D, A3F, A3G, A3H |
| LP1187 | Male | 57 | Venda | Vhembe | 666 | 42 | A3D, A3F, A3G, A3H |
| LP1207 | Female | 53 | Venda | Vhembe | 3454 | 194 | A3D, A3G, A3H |
| LP1225 | Female | 45 | Venda | Vhembe | 310 | 300 | A3D, A3F, A3G, A3H |
| LP1226 | Female | 49 | Venda | Vhembe | 36 | 77 | A3D, A3F, A3G, A3H |
| LP1229 | Female | 50 | Venda | Vhembe | 7671 | 153 | A3D, A3F, A3G, A3H |
| LP1265 | Female | 49 | Venda | Vhembe | 36 | 77 | A3D, A3F, A3G, A3H |
| LP1390 | Male | 53 | Venda | Vhembe | 691 | 386 | A3D, A3F, A3G, A3H |
| LP1436 | Male | 57 | Venda | Vhembe | 65438 | 48 | A3D, A3F, A3G, A3H |
| LP1452 | Male | 46 | Venda | Vhembe | 97865 | 43 | A3D, A3F, A3G, A3H |
| LP1475 | Female | 60 | Venda | Vhembe | 88576 | 699 | - |
| LP1501 | Female | 39 | Venda | Vhembe | 88576 | 699 | A3D, A3F, A3G, A3H |
