# Supplementary material for: Characterization of APOBEC3 variation in a population of HIV-1 infected individuals in northern South Africa
Source: BMC Med Genet. 2019 Jan 19;20:21. doi: 10.1186/s12881-018-0740-4 (PMC6339282; doi:10.1186/s12881-018-0740-4)
Supplement: Supplementary file 2 — Table S2. Apobec 3D- Linkage Disequilibrium Calculations: D’ and R2 values. (PDF 42 kb) [file 12881_2018_740_MOESM2_ESM.pdf]

Table S2

Apobec 3D - LD Calculations
1000G- All Groups

RS_number	**R97C**<br>**75858538**	**T238A**<br>**201709403**	**R248K**<br>**61748819**	T316T<br>184448269
	**D'**	**D'**	**D'**	**D'**
**rs75858538**	1	1	1	1
**rs201709403**	1	1	1	1
**rs61748819**	1	1	1	1
rs184448269	1	1	1	1
	**R^2**	**R^2**	**R^2**	**R^2**
**rs75858538**	1	0	0	0
**rs201709403**	0	1	0	0
**rs61748819**	0	0	1	0.122
rs184448269	0	0	0.122	1

Apobec 3D - LD Calculations
1000G- AFR Group

RS_number	**R97C**<br>**75858538**	**T238A**<br>**201709403**	**R248K**<br>**61748819**	T316T<br>184448269
	**D'**	**D'**	**D'**	**D'**
**rs75858538**	1	1	1	1
**rs201709403**	1	1	1	1
**rs61748819**	1	1	1	1
rs184448269	1	1	1	1
	**R^2**	**R^2**	**R^2**	**R^2**
**rs75858538**	1	0	0.004	0
**rs201709403**	0	1	0	0
**rs61748819**	0.004	0	1	0.099
rs184448269	0	0	0.099	1

The following RS numbers were not found in dbSNP 142: rs772893975, rs61999342, rs769426665
Nonsynonymous SNPs are indicated in **BOLD** font.
