# Supplementary material for: Characterization of APOBEC3 variation in a population of HIV-1 infected individuals in northern South Africa
Source: BMC Med Genet. 2019 Jan 19;20:21. doi: 10.1186/s12881-018-0740-4 (PMC6339282; doi:10.1186/s12881-018-0740-4)
Supplement: Supplementary file 3 — Table S3. Apobec 3F- Linkage Disequilibrium Calculations: D’ and R2 values. (PDF 52 kb) [file 12881_2018_740_MOESM3_ESM.pdf]

Table S3

Apobec 3F - LD Calculations
1000G- All Groups

RS_number	R48P 35053197 D'	A78V 5750728 D'	Q87L 114704208 D'	I87L 146543452 D'	A108S 2020390 D'	R143R 4821862 D'	V231I 2076101 D'	E245E 113109079 D'	Y307C 12157816 D'	*S327S* 35895636 D'
**rs35053197**	1	0.843	1	1	0.874	0.868	0.842	1	1	1
**rs5750728**	0.843	1	1	0.75	0.989	0.994	0.997	1	1	1
**rs114704208**	1	1	1	1	1	1	1	1	1	1
**rs146543452**	1	0.75	1	1	1	0.579	0.748	1	1	1
**rs2020390**	0.874	0.989	1	1	1	0.839	0.996	1	1	1
rs4821862	0.868	0.994	1	0.579	0.839	1	0.995	1	0.07	1
**rs2076101**	0.842	0.997	1	0.748	0.996	0.995	1	1	1	1
**rs113109079**	1	1	1	1	1	1	1	1	1	1
**rs12157816**	1	1	1	1	1	0.07	1	1	1	1
rs35895636	1	1	1	1	1	1	1	1	1	1

	R^2	R^2	R^2	R^2	R^2	R^2	R^2	R^2	R^2	R^2
**rs35053197**	1	0.007	0	0	0.007	0.011	0.007	0	0	0
**rs5750728**	0.007	1	0.015	0.001	0.854	0.677	0.975	0.003	0.017	0.004
**rs114704208**	0	0.015	1	0	0.018	0.022	0.015	0	0	0
**rs146543452**	0	0.001	0	1	0.001	0.001	0.001	0	0	0
**rs2020390**	0.007	0.854	0.018	0.001	1	0.552	0.85	0.003	0.019	0.005
rs4821862	0.011	0.677	0.022	0.001	0.552	1	0.666	0.004	0	0.003
**rs2076101**	0.007	0.975	0.015	0.001	0.85	0.666	1	0.003	0.016	0.004
**rs113109079**	0	0.003	0	0	0.003	0.004	0.003	1	0	0
**rs12157816**	0	0.017	0	0	0.019	0	0.016	0	1	0
rs35895636	0	0.004	0	0	0.005	0.003	0.004	0	0	1

Apobec 3F - LD Calculations
1000G- AFR Group

RS_number	R48P 35053197 D'	A78V 5750728 D'	Q87L 114704208 D'	I87L 146543452 D'	A108S 2020390 D'	R143R 4821862 D'	V231I 2076101 D'	E245E 113109079 D'	Y307C 12157816 D'	S327S 35895636 D'
**rs35053197**	1	1	1	1	1	1	1	1	1	1
**rs5750728**	1	1	1	0.381	0.956	0.984	0.995	1	1	1
**rs114704208**	1	1	1	1	1	1	1	1	1	1
**rs146543452**	1	0.381	1	1	1	0.542	0.331	1	1	1
**rs2020390**	1	0.956	1	1	1	0.157	0.994	1	1	1
rs4821862	1	0.984	1	0.542	0.157	1	0.991	1	1	1
**rs2076101**	1	0.995	1	0.331	0.994	0.991	1	1	1	1
**rs113109079**	1	1	1	1	1	1	1	1	1	1
**rs12157816**	1	1	1	1	1	1	1	1	1	1
rs35895636	1	1	1	1	1	1	1	1	1	1

	R^2	R^2	R^2	R^2	R^2	R^2	R^2	R^2	R^2	R^2
**rs35053197**	1	0.008	0.002	0	0.067	0.038	0.007	0	0.001	0.001
**rs5750728**	0.008	1	0.015	0	0.483	0.204	0.899	0.003	0.009	0.004
**rs114704208**	0.002	0.015	1	0	0.028	0.07	0.013	0.001	0.002	0.001
**rs146543452**	0	0	0	1	0.013	0.002	0	0	0	0
**rs2020390**	0.067	0.483	0.028	0.013	1	0.01	0.474	0.022	0.017	0.008
rs4821862	0.038	0.204	0.07	0.002	0.01	1	0.188	0.013	0.03	0.014
**rs2076101**	0.007	0.899	0.013	0	0.474	0.188	1	0.002	0.008	0.004
**rs113109079**	0	0.003	0.001	0	0.022	0.013	0.002	1	0	0
**rs12157816**	0.001	0.009	0.002	0	0.017	0.03	0.008	0	1	0.001
rs35895636	0.001	0.004	0.001	0	0.008	0.014	0.004	0	0.001	1

The following RS number(s) were not found in dbSNP 142: Y196Y (rs765418322). Also no results were obtained for S118S (rs35928287), and S229S (rs549550231).
