# Supplementary material for: Characterization of APOBEC3 variation in a population of HIV-1 infected individuals in northern South Africa
Source: BMC Med Genet. 2019 Jan 19;20:21. doi: 10.1186/s12881-018-0740-4 (PMC6339282; doi:10.1186/s12881-018-0740-4)
Supplement: Supplementary file 4 — Table S4. Apobec 3G- Linkage Disequilibrium Calculations: D’ and R2 values. (PDF 33 kb) [file 12881_2018_740_MOESM4_ESM.pdf]

Table S4

Apobec 3G - LD Calculations
1000G- All Groups

	S60S	F119F	**H186R**	**R256H**	**Q275E**	**G363R**	L371L
RS_number	1.13E+08	5757465	**8177832**	**17000736**	**17496046**	**1.48E+08**	11545130
	**D'**	**D'**	**D'**	**D'**	**D'**	**D'**	**D'**
rs112603901	1	1	1	1	1	1	1
rs5757465	1	1	1	1	1	1	1
**rs8177832**	1	1	1	1	1	1	1
**rs17000736**	1	1	1	1	1	1	1
**rs17496046**	1	1	1	1	1	1	1
**rs148267053**	1	1	1	1	1	1	1
rs11545130	1	1	1	1	1	1	1
	**R^2**	**R^2**	**R^2**	**R^2**	**R^2**	**R^2**	**R^2**
rs112603901	1	0	0	0	0	0	0
rs5757465	0	1	0.068	0.002	0.024	0.002	0.003
**rs8177832**	0	0.068	1	0.001	0.01	0.001	0.001
**rs17000736**	0	0.002	0.001	1	0	0	0
**rs17496046**	0	0.024	0.01	0	1	0	0
**rs148267053**	0	0.002	0.001	0	0	1	0
rs11545130	0	0.003	0.001	0	0	0	1

Apobec 3G - LD Calculations
1000G- AFR Group

	S60S	F119F	**H186R**	**R256H**	**Q275E**	**G363R**	L371L
RS_number	1.13E+08	5757465	**8177832**	**17000736**	**17496046**	**1.48E+08**	11545130
	**D'**	**D'**	**D'**	**D'**	**D'**	**D'**	**D'**
rs112603901	1	1	1	1	1	1	1
rs5757465	1	1	1	1	1	1	1
**rs8177832**	1	1	1	1	1	1	1
**rs17000736**	1	1	1	1	1	1	1
**rs17496046**	1	1	1	1	1	1	1
**rs148267053**	1	1	1	1	1	1	1
rs11545130	1	1	1	1	1	1	1
	**R^2**	**R^2**	**R^2**	**R^2**	**R^2**	**R^2**	**R^2**
rs112603901	1	0	0.002	0	0	0	0
rs5757465	0	1	0.022	0	0.004	0	0.001
**rs8177832**	0.002	0.022	1	0.011	0.108	0.011	0.021
**rs17000736**	0	0	0.011	1	0.002	0	0
**rs17496046**	0	0.004	0.108	0.002	1	0.002	0.004
**rs148267053**	0	0	0.011	0	0.002	1	0
rs11545130	0	0.001	0.021	0	0.004	0	1

No results were obtained for: A109A (rs375760983). Nonsynonymous SNPs are indicated in **BOLD**
