# Supplementary material for: Characterization of APOBEC3 variation in a population of HIV-1 infected individuals in northern South Africa
Source: BMC Med Genet. 2019 Jan 19;20:21. doi: 10.1186/s12881-018-0740-4 (PMC6339282; doi:10.1186/s12881-018-0740-4)
Supplement: Supplementary file 5 — Table S5. Apobec 3H- Linkage Disequilibrium Calculations: D’ and R2 values. (PDF 50 kb) [file 12881_2018_740_MOESM5_ESM.pdf]

Table S5

Apobec 3H - LD Calculations
1000G- All Groups

RS_number	**N15Δ 140936762**	**R18K 139293**	T43T 139294	**G105R 139297**	**K121E 139298**	**K140E 139300**	**E178D 139302**
	**D'**	**D'**	**D'**	**D'**	**D'**	**D'**	**D'**
**rs140936762**	1	1	0.995	0.962	0.961	NA	0.922
**rs139293**	1	1	0.992	0.952	0.952	NA	0.931
rs139294	0.995	0.992	1	0.973	0.973	NA	0.934
**rs139297**	0.962	0.952	0.973	1	1	NA	0.984
**rs139298**	0.961	0.952	0.973	1	1	NA	0.984
**rs139300**	NA	NA	NA	NA	NA	NA	NA
**rs139302**	0.922	0.931	0.934	0.984	0.984	NA	1
	**R^2**	**R^2**	**R^2**	**R^2**	**R^2**	**R^2**	**R^2**
**rs140936762**	1	0.543	0.434	0.39	0.382	NA	0.372
**rs139293**	0.543	1	0.234	0.207	0.203	NA	0.206
rs139294	0.434	0.234	1	0.91	0.891	NA	0.87
**rs139297**	0.39	0.207	0.91	1	0.98	NA	0.933
**rs139298**	0.382	0.203	0.891	0.98	1	NA	0.914
**rs139300**	NA	NA	NA	NA	NA	NA	NA
**rs139302**	0.372	0.206	0.87	0.933	0.914	NA	1

Apobec 3H - LD Calculations
1000G- AFR Group

RS_number	**N15Δ 140936762**	**R18K 139293**	T43T 139294	**G105R 139297**	**K121E 139298**	**K140E 139300**	**E178D 139302**
	**D'**	**D'**	**D'**	**D'**	**D'**	**D'**	**D'**
**rs140936762**	1	1	1	1	1	NA	0.678
**rs139293**	1	1	1	1	1	NA	1
rs139294	1	1	1	0.993	0.993	NA	0.773
**rs139297**	1	1	0.993	1	1	NA	1
**rs139298**	1	1	0.993	1	1	NA	1
**rs139300**	NA	NA	NA	NA	NA	NA	NA
**rs139302**	0.678	1	0.773	1	1	NA	1
	**R^2**	**R^2**	**R^2**	**R^2**	**R^2**	**R^2**	**R^2**
**rs140936762**	1	0.161	0.092	0.063	0.063	NA	0.037
**rs139293**	0.161	1	0.015	0.01	0.01	NA	0.013
rs139294	0.092	0.015	1	0.674	0.674	NA	0.517
**rs139297**	0.063	0.01	0.674	1	1	NA	0.791
**rs139298**	0.063	0.01	0.674	1	1	NA	0.791
**rs139300**	NA	NA	NA	NA	NA	NA	NA
**rs139302**	0.037	0.013	0.517	0.791	0.791	NA	1

NA= not calculated. Nonsynonymous SNPs are indicated in **BOLD** font.
